# Alternative polyadenylation factors link cell cycle to migration

**DOI:** 10.1186/s13059-018-1551-9

**Published:** 2018-10-25

**Authors:** Mithun Mitra, Elizabeth L Johnson, Vinay S Swamy, Lois E Nersesian, David C Corney, David G Robinson, Daniel G Taylor, Aaron M Ambrus, David Jelinek, Wei Wang, Sandra L Batista, Hilary A Coller

**Affiliations:** 1 Department of Molecular, Cell and Developmental Biology, University of California, Los Angeles, Los Angeles, CA USA; 2 Department of Biological Chemistry, David Geffen School of Medicine, University of California, Los Angeles, CA USA; 3 Department of Molecular Biology, Princeton University, Princeton, NJ USA; 4 Department of Biochemistry, University of California, Los Angeles, Los Angeles, CA USA; 5 Department of Chemical Engineering, University of California, Los Angeles, Los Angeles, CA USA; 6 Lewis-Sigler Institute for Integrative Genomics, Princeton University, Princeton, NJ USA; 7 Department of Computer Science, University of Southern California, Los Angeles, CA USA

**Keywords:** mRNA processing, Proliferation, Quiescence, Migration, Wound healing

## Abstract

**Background:**

In response to a wound, fibroblasts are activated to migrate toward the wound, to proliferate and to contribute to the wound healing process. We hypothesize that changes in pre-mRNA processing occurring as fibroblasts enter the proliferative cell cycle are also important for promoting their migration.

**Results:**

RNA sequencing of fibroblasts induced into quiescence by contact inhibition reveals downregulation of genes involved in mRNA processing, including splicing and cleavage and polyadenylation factors. These genes also show differential exon use, especially increased intron retention in quiescent fibroblasts compared to proliferating fibroblasts. Mapping the 3′ ends of transcripts reveals that longer transcripts from distal polyadenylation sites are more prevalent in quiescent fibroblasts and are associated with increased expression and transcript stabilization based on genome-wide transcript decay analysis. Analysis of dermal excisional wounds in mice reveals that proliferating cells adjacent to wounds express higher levels of cleavage and polyadenylation factors than quiescent fibroblasts in unwounded skin. Quiescent fibroblasts contain reduced levels of the cleavage and polyadenylation factor CstF-64. CstF-64 knockdown recapitulates changes in isoform selection and gene expression associated with quiescence, and results in slower migration.

**Conclusions:**

Our findings support cleavage and polyadenylation factors as a link between cellular proliferation state and migration.

**Electronic supplementary material:**

The online version of this article (10.1186/s13059-018-1551-9) contains supplementary material, which is available to authorized users.

## Background

Fibroblasts within the dermis bear much of the responsibility for the secretion and maintenance of extracellular matrix proteins [1]. Fibroblasts in unwounded skin are mostly in a state of quiescence in which they have reversibly exited the proliferative cell cycle [1–3]. In the initial response to a wound, mitogens and chemokines such as platelet-derived growth factor and fibroblast growth factor released by platelets and keratinocytes stimulate fibroblasts to migrate to the wound-healing environment and proliferate [1–4]. In the wounded tissue, fibroblasts secrete collagen and other extracellular matrix molecules that remodel the extracellular environment and promote the formation of a scar [3]. While fibroblasts are recognized to play an important role in normal skin and in the wound-healing environment, we do not yet have a full appreciation of the molecular mechanisms that control the changes in fibroblast behavior in the context of a wound.

We have been studying the transition between proliferation and quiescence in a model system in primary human dermal fibroblasts [5–9]. Using microarrays, we and others have shown that a shift between proliferation and quiescence is associated with a major reprogramming of gene expression patterns, and that these gene expression changes are important for the functional attributes of quiescent cells, such as their ability to re-enter the cell cycle [9–12]. Based on our previous studies showing changes in the levels of splicing factors as fibroblasts transition between proliferation and quiescence [9], and earlier studies showing that proliferating cells, stem cells, activated cells, and cancer cells rely heavily on alternative polyadenylation (APA) by preferential use of proximal polyadenylation sites [13–21], we sought to understand whether alternative isoform use [16, 22, 23] could represent a link between proliferation and migration.

To address this question, we defined the changes in isoform use and polyadenylation site selection that occur as cells transition from proliferation to quiescence. We found that APA factors are expressed at lower levels as fibroblasts become quiescent, and that knockdown of these factors results in APA and gene expression changes that overlap with the changes that occur with quiescence. Longer transcripts that end at distal polyadenylation sites tend to be more stable than shorter transcripts generated from proximal polyadenylation site use in proliferating cells. We also discovered that APA factors are functionally important for the transition to a more migratory state in proliferating versus quiescent fibroblasts and affect migration in cancer cells as well. Our data, taken as a whole, provide a deeper understanding of the role of mRNA processing in the close association between proliferation and migration.

## Results

### Entry into quiescence results in downregulation of genes involved in the cell cycle, mRNA processing, and motility

**Fig. 1:**
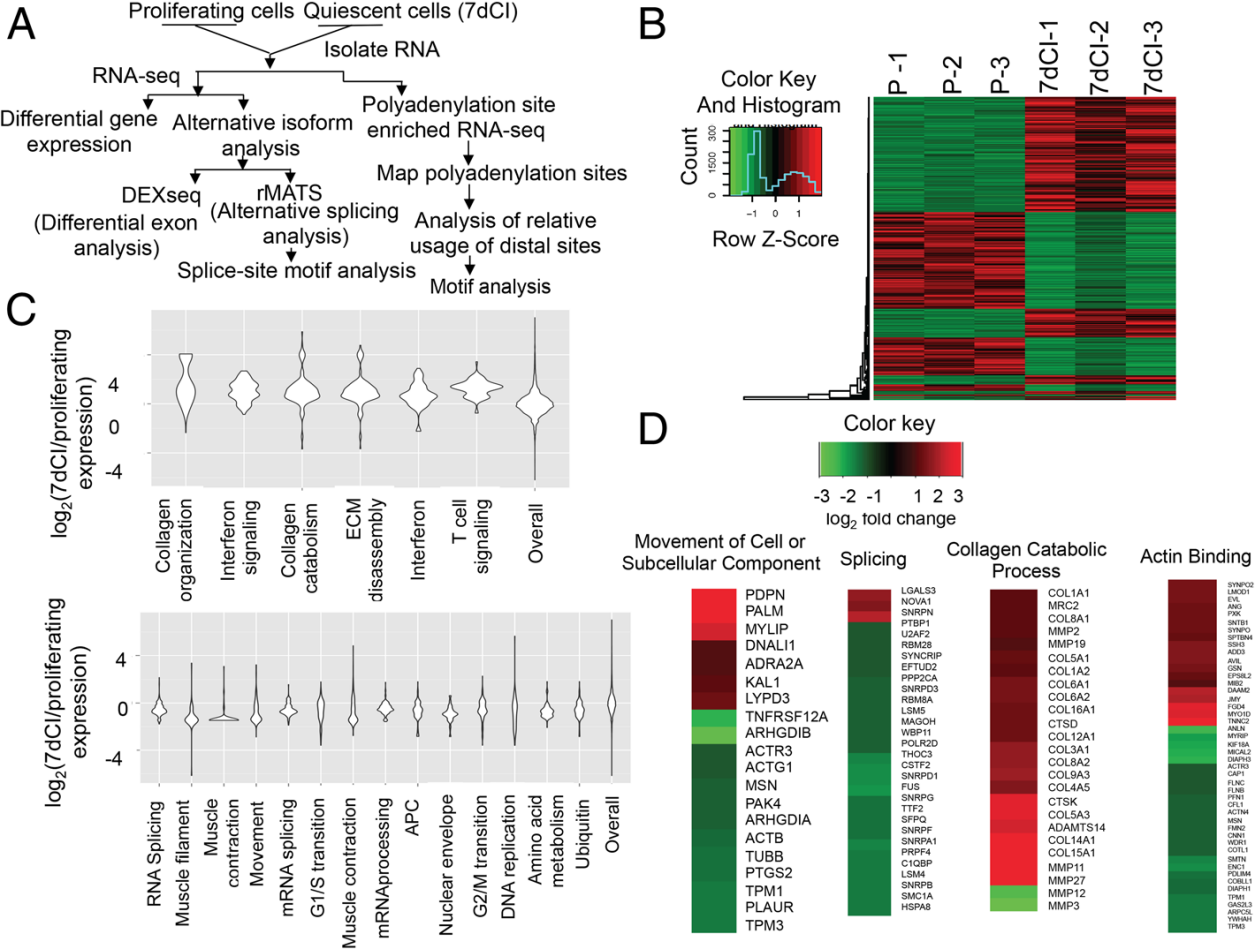
RNA-Seq analysis of gene expression changes in proliferating versus quiescent fibroblasts. **a** Schematic of RNA-Seq-based analysis of proliferating and quiescent fibroblasts performed in this study. **b** Total RNA was isolated from three independent biological replicates of proliferating fibroblasts and three matched independent biological replicates of 7dCI fibroblasts. RNA samples were converted to cDNA libraries and sequenced on an Illumina Hi-Seq 2000. Reads were aligned to the human genome (hg19 human reference sequence) and the number of reads mapping to each gene (UCSC gene annotation) in the genome was determined. A heatmap of read counts for 1993 genes with at least a twofold change in expression and a false discovery rate (FDR) < 5% is shown. Hierarchical clustering is denoted by the dendrogram to the left of the heatmap. A color key and a histogram displaying the density of genes at a given color intensity are shown in the upper left corner. **c** Gene set enrichment analysis was used to determine the gene sets most significantly upregulated (top) or downregulated (bottom) with quiescence. Gene sets are listed in descending order of statistical significance from left to right. A histogram of the log_2_(fold-change) of the normalized read count in 7dCI compared to proliferating fibroblasts for each gene in the gene set is plotted in a violin plot representation. **d** Heat maps of genes within selected gene set enrichment categories are provided. The log_2_ ratio of normalized RNA-Seq counts in 7dCI compared with proliferating fibroblasts are shown. Red indicates higher expression in quiescent than proliferating fibroblasts; green indicates higher expression in proliferating than quiescent fibroblasts. Only genes in each category that change in expression two-fold or more are included

Primary human dermal fibroblasts were isolated from human skin samples as previously described [24]. Fibroblasts isolated from two different donors were collected in proliferating conditions or after being induced into quiescence by 7 days of contact inhibition (7dCI) of proliferation [7]. RNA-Seq and microarray analyses were performed to determine changes in gene expression between three samples of proliferating and matched 7dCI cells (Fig. 1a and Additional file 1: Table S1) [25]. Among the 19,673 genes monitored, transcripts from 1993 genes (10.1%) changed in expression twofold or more, demonstrating widespread changes in gene expression with contact inhibition-induced quiescence (Fig. 1b). Expression levels for 52% of these genes were upregulated in 7dCI compared with proliferating fibroblasts, and 48% were downregulated in 7dCI fibroblasts. Correlation between biological replicates analyzed by RNA-Seq was high (*R*^2^ values greater than or equal to 0.83) (Additional file 1: Figure S1A). When the same samples were analyzed with microarrays, the differential gene expression detected by microarray was largely in agreement with that detected by RNA-Seq (*r*^2^ = 0.785, *p* < 0.001) (Additional file 1: Figure S1B). Further, gene expression changes detected by RNA-Seq correlated well with the previously published “quiescence program” of gene expression changes identified in fibroblasts induced into quiescence by multiple independent conditions [9] (Additional file 1: Figure S1C). The findings support previous studies showing that quiescence is associated with regulation of a significant fraction of the genome [9, 10, 26].

Gene set enrichment analysis (GSEA) [27, 28] revealed that expression of genes involved in DNA replication and cell cycle regulation was downregulated in 7dCI compared with proliferating fibroblasts (Fig. 1c), consistent with cell cycle exit in contact-inhibited conditions. Expression of genes associated with extracellular matrix remodeling and collagen metabolism was upregulated with quiescence (Fig. 1c, d), consistent with our previous findings [6, 7]. Indeed, COL21A1, a collagen found associated with collagen I, is among the genes most strongly induced in quiescent compared with proliferating fibroblasts (Additional file 1: Table S2). Expression of genes in the categories of muscle filament sliding, regulation of muscle contraction, movement, and muscle contraction was downregulated in contact-inhibited compared with proliferating fibroblasts (Fig. 1c, d). Four genes involved in cell motility were among the most strongly downregulated genes with quiescence (KISS1, ACTC1, PODXL, and RLTPR) (Table 1 and Additional file 1: Table S2). Thus, we found that proliferating fibroblasts express higher levels of transcripts associated with motility and cytoskeletal remodeling.

**Table 1:** List of genes involved in motility that are altered with quiescence

Type of change (analysis)	Genes
Differential expression (DESeq)	KISS1, ACTC1, PODXL, RLTPR, PDPN, PALM, MYLIP, DNALI1, ADRA2A, KAL1, LYPD3, TNFRSF12A, ARHGDIB, ACTR3, ACTG1, MSN, PAK4, ARHGDIA, ACTB, TUBB, PTGS2, TPM1, PLAUR, TPM3
Alternative splicing (rMATS)	FYN, HGF, CD44, MAP4, ATP2B4, MYLK, ACTN1, SCARB1, FGFR1, FAT1, MYL6, TSPO, AKT2, BRAT1, ARHGAP21, GTPBP4, SOD2, FN1, WIPF1, CD58, KIF17, ITGB1BP1, GLIPR2, CALD1, LRP1, KLC1, KTN1, CHN1, POSTN, NUMB, ENPP2, KIF13A, TPM1, CLTC, MINK1, SH3KBP1, CACNA1C, DAB2, THY1, NCK1, SHC1, NFASC, IL17RC, LAMB2, PTPRM, GAS6, CCDC125, LAMA2, FLNA, SPTAN1, TPM2, CDC42BPB
UTR-type alternative polyadenylation for 2pA site-containing genes	MACF1, LMNA, DDR2, LAMC1, NAV1, PEX13, ACTR2, IL1R1, MYO1B, SP100, TRAK1, NCK1, OPA1, PDGFRA, AP3S1, TUBB, PTP4A1, FGFR1OP, ANLN, GLIPR2, AMOTL1, PTPN11, MMP14, FRMD6, SMAD3, DYNLL2, PTPRA, DOK5, COL18A1, SSX2IP, NRAS, CERS2, SDC1, DPP4, DYNC1LI1, MAP4, WNT5A, CD47, DPYSL3, LAMA4, ARHGAP18, CITED2, GNA12, STC1, EXT1, AP3M1, BLOC1S2, RRAS2, TBX5, SYNJ2BP, EMP2, AMFR, STAT3, COL1A1, CCBE1, AP3D1, SDC4, APP, RBFOX2, MYH9, RAP2C
Upstream region-type alternative polyadenylation for 2 pA site-containing genes	RDX, PEX14, DYNC2LI1, SUN1, RECK, IQGAP1, DOCK7

Transcripts associated with splicing and polyadenylation were mostly downregulated in 7dCI compared with proliferating fibroblasts (Fig. 1c, d), consistent with previous reports [9, 21]. Transcripts encoding many of the proteins that are considered core components of the spliceosome were slightly downregulated in contact-inhibited compared with proliferating fibroblasts (Additional file 1: Table S3), with three genes reaching statistical significance (U1C (2.26-fold reduction), PRPF4 (2.77-fold reduction), and PPIH (2.89-fold reduction)). Expression levels of cleavage and polyadenylation factors were also reduced with quiescence (Additional file 2). We hypothesized that in addition to changes in gene expression, alterations in mRNA processing events between proliferating and quiescent fibroblasts could also contribute to functional changes in quiescent and proliferating states.

### Quiescent fibroblasts retain more exons and introns than proliferating fibroblasts

To better understand changes in mRNA processing associated with proliferation, we investigated our RNA-Seq data further to identify examples of alternative start site, alternative splicing, or alternative polyadenylation. Applying the DEXSeq algorithm [29], we discovered 1975 exons, encoded within 1218 genes, with differential expression between proliferating and 7dCI fibroblasts (Additional file 3). Using g:Profiler [30], we found that genes that undergo alternative isoform expression in proliferating versus quiescent cells are enriched in categories of RNA binding, RNA processing, translational elongation, and RNA splicing (Table 2, Additional file 4). Thus, genes involved in RNA processing are themselves particularly likely to be alternatively processed during the transition between proliferation and quiescence.

**Table 2:** List of splicing genes undergoing differential pre-mRNA processing with quiescence

Type of pre-mRNA processing (analysis)	Splicing genes
Differential exon use (DEXSeq)	KDM1A, LUC7L, AQR, ZCCHC8, SFSWAP, U2AF2, SUGP2, SNRPA, YTHDC1, FUS, PAPOLA, HNRNPC, HNRNPH3, HNRNPM, POLR2E, SNU13, RBFOX2, SRSF5, ACIN1, PABPC1L, USB1, SNRNP70, RBM28, LSM5, CASC3, LUC7L3, DHX15, MAGOHB, SRSF9, SRSF3, PAPOLG, SF3B1, SRSF7, SRSF4, SFPQ, SRSF11, RBM25, DDX39A, SRSF6, RBM39, SNRPA1, ZRANB2, SRRM1, RBM17, CCNH, RBMX2, TRA2B, HNRNPD, SCAF11, SNRPG, SNRNP200, SREK1, RNF20, CCAR2, U2AF1, CHTOP, ZNF326, TRA2A, CDK12, SF1, HNRNPH1, RBM4B, RSRC1, GEMIN4, NPM1, SF3A3, PRPF39, ADARB1, UBL5
Alternative splicing (rMATS)	LUC7L, ZCCHC8, SRRT, HNRNPC, HNRNPH3, NHP2L1, DDX17, RBM23, PRMT5, SRSF5, ACIN1, PQBP1, SNRNP70, POLR2I, DDX5, MAGOHB, SRSF3, PRPF4B, SF3B1, SRSF7, TIA1, SRSF11, RBM25, DDX39A, SRSF6, SNRPB, DNAJC8, RBM39, SNRPA1, SRRM1, PNPT1, HNRNPD, SCAF11, MBNL1, SREK1, AFF2, RBPMS, U2AF1, CHTOP, SRSF2, TRA2A, HNRNPH1, PRPF39, UBL5, DDX39B
UTR-type alternative polyadenylation for 2pA site-containing genes	WDR77, SNRNP40, SF3A3, CHTOP, TSEN15, KHDRBS1, RBM17, HNRNPH3, CSTF3, RBM4B, MAGOHB, SART3, CMTR2, TXNL4B, POLR2C, SF3B3, SRSF2, RNMT, GTF2F1, KHSRP, HNRNPUL1, PTBP1, CIR1, TIA1, GCFC2, APP, RBFOX2, NHP2L1, POLDIP3, ISY1, NCBP2, RSRC1, POLR2H, PAPD4
Upstream region-type alternative polyadenylation for 2 pA site-containing genes	HNRNPR, WBP11, RBBP6, SAFB2, PRPF4B, PSIP1, ZRSR2

**Fig. 2:**
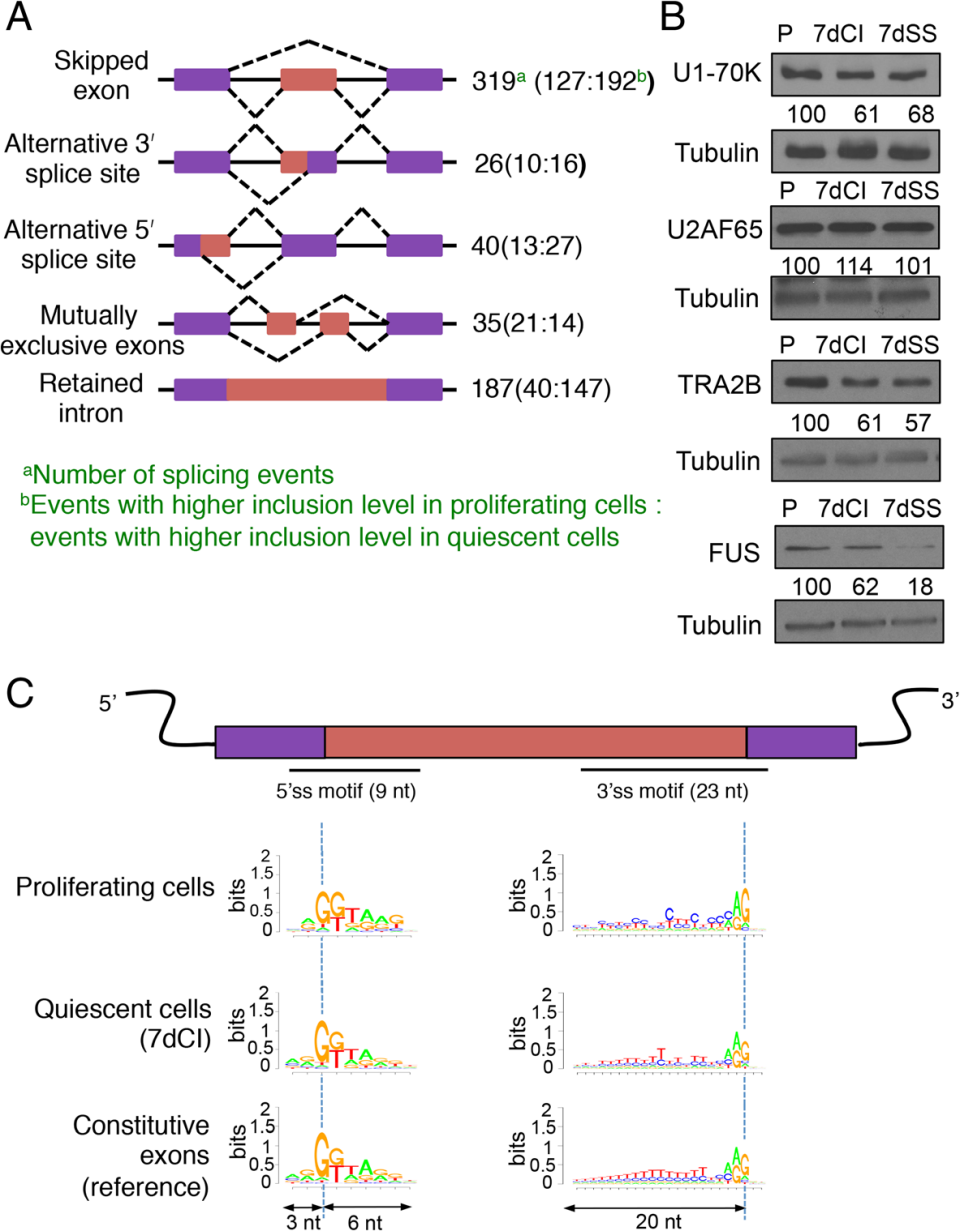
Differential splicing in proliferating and quiescent fibroblasts. **a** rMATS was applied to RNA-Seq data from three biological replicates of proliferating fibroblasts and three biological replicates of contact-inhibited fibroblasts. Splicing events with an FDR < 0.05 are shown. The total numbers of splicing events are reported. In parentheses, the number of events with higher inclusion in proliferating fibroblasts is provided, followed by the number of events with higher inclusion in quiescent fibroblasts. Skipped exons were significantly more likely to be included in quiescent fibroblasts (Fisher’s exact test, two-tailed *p* value = 0.013). Introns were significantly more likely to be retained in quiescent fibroblasts (Fisher’s exact test, two-tailed *p* value < 0.0001). **b** Immunoblotting of splicing factors in proliferating and quiescent fibroblasts. Levels of core splicing factor U2AF65 were similar in proliferating and quiescent fibroblasts. U1-70 K and auxiliary factors TRA2β and FUS were expressed at lower levels in 7dCI and 7dSS compared with proliferating fibroblasts. α-Tubulin was analyzed as a loading control. The ratio of splicing factor to tubulin, normalized to proliferating cells, is shown below. **c** Sequence logos [120] are provided for 5′ and 3′ sequences for exons that are constitutively spliced, and introns that are preferentially retained in proliferating or quiescent cells. The *y*-axis indicates bits of information [121]. 3′ splice site sequences were different between proliferating versus constitutive conditions (*p* value < 0.01 for constitutive versus retained in proliferating conditions, ANOVA with Tukey’s multiple comparison test) and quiescent versus constitutive conditions (*p* value < 0.01 for constitutive versus retained in quiescent conditions)

To better understand the frequency of specific types of splicing events that occurred differentially in proliferating and quiescent fibroblasts, we applied the rMATS computational algorithm [31–33] (Fig. 2a, Additional file 5). Skipped exons (exons that are present in proliferating, but not quiescent, cells or vice versa) were the most common type of event detected (319 events, 53% of events). Of the splicing events detected by rMATS, 39% were also detected by DEXSeq. More exons were preferentially included in quiescent compared with proliferating conditions, than proliferating compared with quiescent conditions (1.5-fold, Fisher’s exact test, two-tailed *p* value = 0.013) (Fig. 2a). These exon-switching events provide opportunities for regulation of protein function based on the inclusion or exclusion of individual exons. Introns were significantly more frequently retained in quiescent than proliferating fibroblasts (3.7-fold, Fisher’s exact test, two-tailed *p* value < 0.0001) (Fig. 2a). 8.2% of the transcripts associated with retained intron events are annotated as nonsense-mediated decay (NMD) candidates (18 unique NMD transcripts/220 total unique intron retention transcripts in the Ensembl database). Gene ontology (GO) analysis of the differentially spliced genes revealed that genes that undergo alternative splicing with quiescence are enriched for the categories of RNA binding, RNA processing, and RNA splicing (Table 2 and Additional file 6), consistent with a growing literature demonstrating that genes involved in mRNA splicing are themselves regulated by splicing events [30, 34–37].

### Some auxiliary splicing factors are downregulated in quiescent fibroblasts

To understand the changes in splicing in quiescent compared with proliferating fibroblasts, we investigated changes in the expression of splicing factors. Our RNA-Seq data revealed that expression from RNA splicing genes is modestly downregulated in contact-inhibited fibroblasts (Fig. 1c, d and Additional file 1: Table S3). We monitored protein levels of splicing factors with immunoblotting in fibroblasts that were proliferating or induced into quiescence by 7 days of contact inhibition (7dCI) or by serum starvation (7dSS). Levels of essential splicing factor U2AF65 were similar in proliferating and quiescent fibroblasts. Levels of core factor U1-70K and auxiliary factors TRA2β and FUS were downregulated in quiescent compared with contact-inhibited fibroblasts (Fig. 2b). Lower levels of some splicing factors in quiescent fibroblasts may contribute to the increased intron retention in quiescent conditions [38, 39].

### Weaker splice sites for retained introns

In addition to lower levels of splicing factors, intron retention has been associated with weak splice sites [40, 41]. To better understand why some introns are retained in proliferating or quiescent cells, we analyzed the extent to which 5′ splice sites (9-nt length) and 3′ splice sites (23 nt) of differentially retained introns match consensus splice sites [42]. We determined the probability of observing each sequence given the position weight matrix for consensus splice sites. Sequences at splice sites for introns differentially retained in proliferating or quiescent states matched the consensus sequence less well than the sequences near constitutively spliced exons, with a strong effect at the 3′ splice site (Fig. 2c). These findings are consistent with previous studies that also showed that 3′ splice sites are enriched for C’s compared with T’s in the polypyrimidine tracts of introns that are retained [43]. Thus, in proliferating fibroblasts that have higher levels of most splicing factors, intron retention may be especially sensitive to the 3′ splice sequence.

### A shift toward the use of more distal polyadenylation sites in quiescence

**Fig. 3:**
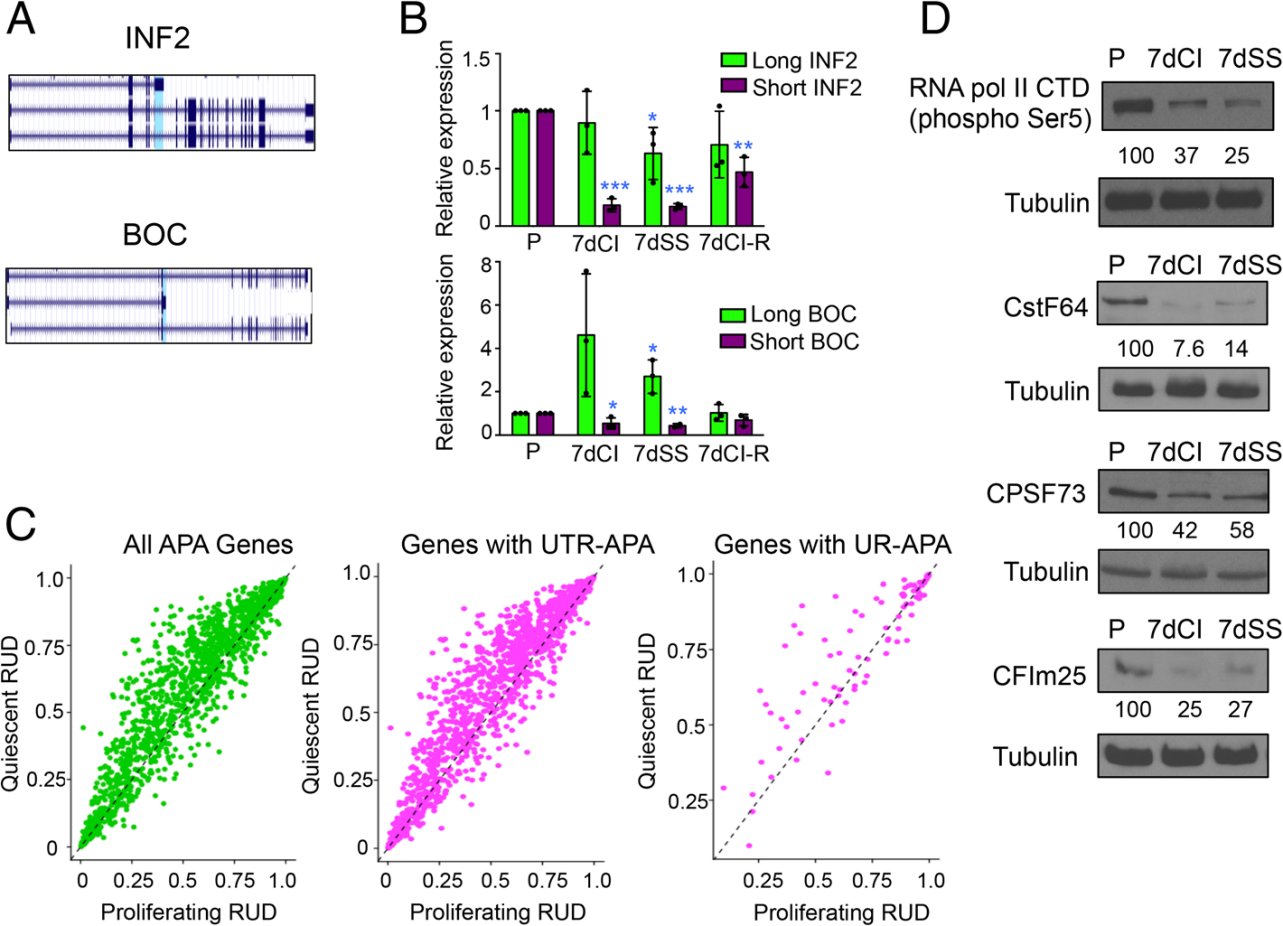
Use of distal polyadenylation sites and lower levels of cleavage and polyadenylation factors during quiescence. **a** UCSC Genome browser views showing the long and short isoforms of INF2 and BOC. The differentially expressed exon is highlighted in cyan. **b** Real-time PCR validation of APA with quiescence. cDNA samples generated from fibroblasts that were proliferating, quiescent by contact inhibition or serum starvation, or induced into quiescence by serum-starvation and then restimulated, were analyzed with real-time PCR. Primers were designed to recognize the short (terminating at the proximal polyadenylation site) or long (terminating at the distal polyadenylation site) isoforms of INF2 or BOC. Transitioning fibroblasts into quiescence resulted in reduced expression of the short isoform of INF2 and increased expression of the long isoform of BOC. Restimulating quiescent fibroblasts resulted in expression patterns of the short and long isoforms that more closely resemble proliferating cells. Plots show individual datapoints as dots. Bar graphs represent mean and average ± S.D. The number of replicates for all conditions for short and long INF2 is 3. The number of replicates for all conditions for long BOC is 3. The number of replicates for P, 7dCI, and 7dCI-R for short BOC is 3. The number of replicates for 7dSS for short BOC is 2. Statistical significance in knockdown cells compared to control cells was determined for long and short isoforms with two-tailed, unpaired *t* tests. For all figures, one asterisk indicates *p* value < 0.05. Two asterisks indicate *p* value < 0.01. Three asterisks indicate *p* value < 0.001. **c** A shift toward expression of longer isoforms in quiescent fibroblasts. Proliferating and 7dCI were analyzed by polyadenylation site-enriched RNA-Seq. Relative use of the distal polyadenylation site (RUD) for individual genes in proliferating fibroblasts is plotted on the x-axis and RUD for the same gene in quiescent conditions is plotted on the y-axis. The dashed black line indicates *y* = *x*. The first plot (left) displays all genes with two detected polyadenylation sites. The middle plot displays UTR APA genes and the final plot (right) shows the same data for genes that undergo UR APA. **d** Immunoblotting was performed on protein lysates collected from proliferating, 7dCI and 7dSS fibroblasts for CstF-64, CFIm25, and CPSF73. Phosphorylation of serine 5 on RNA pol II CTD was monitored by immunoblotting and levels decline with quiescence. α-Tubulin was monitored as a loading control

A shift toward the use of distal polyadenylation sites has been observed in previous studies that showed that non-dividing cells [21] and differentiated cells [18, 20, 44, 45] predominantly use distal polyadenylation sites, while proliferating cells [18, 21] and cancer cell lines [20, 45, 46] tend to use proximal polyadenylation sites. Our DEXSeq analysis revealed that many of the changes in isoform expression detected between proliferating and 7dCI fibroblasts involve the last exon of the analyzed transcript and would result in a change in polyadenylation site. For example, Inverted Formin, FH2 and WH2 domain (INF2), and brother of CDO (BOC) (Fig. 3a) exhibit alternative use of terminal exons in proliferating and 7dCI fibroblasts. Real-time PCR with isoform-specific primers confirmed that for both INF2 and BOC, the transition to quiescence in response to either 7dCI or 7dSS resulted in a change in polyadenylation site selection (Fig. 3b). For INF2, the strongest effect was a decrease in the use of the proximal polyadenylation site. For BOC, the strongest effect was an increase in the use of the distal polyadenylation site in quiescent fibroblasts. Restimulation of 7dCI fibroblasts to a proliferative state resulted in a reversal back toward a polyadenylation site selection profile more similar to that in proliferating cells for both INF2 and BOC.

To generate a large-scale dataset that would clearly define the 3′ ends of transcripts in proliferating and quiescent (7dCI) fibroblasts, we applied polyadenylation site-enriched RNA-Seq [47]. With polyadenylation site-enriched RNA-Seq, ~ 64% of all mapped sequencing reads matched a polyadenylation site (Additional file 1: Table S4). Polyadenylation site-enriched RNA-Seq data were used to determine the relative use of the distal (RUD) (reads mapping to the distal polyadenylation site/total reads from proximal and distal polyadenylation sites) for each gene in proliferating and 7dCI conditions for detected genes with two polyadenylation sites (Additional file 7). For genes with greater than two polyadenylation sites (Additional file 8), a more general parameter called relative site usage (reads mapping to a polyadenylation site/total reads from all polyadenylation sites) was used. Data were highly reproducible when different biological replicates of proliferating and 7dCI samples were compared (Additional file 1: Figure S2A). Using polyadenylation site-enriched RNA-Seq, we confirmed the previous finding [21] of a shift toward the use of more distal polyadenylation sites upon entry into the quiescent state through contact inhibition (Fig. 3c, Additional file 7). Eighty-eight percent (628 out of 714) of genes with two polyadenylation sites, and with significant changes (|RUD| > 0.05) in alternative polyadenylation (APA) between the two cell states, were longer (greater use of distal pA sites compared to proximal pA sites) in the quiescent compared with the proliferating fibroblasts. For 572 of these 628 genes (91%), the proximal polyadenylation site localizes to the 3′ untranslated region (UTR; termed as UTR APA) (Fig. 3c), while for the remaining 9% of genes, the proximal polyadenylation site is found in the region upstream of the 3´ UTR (upstream region (UR) APA) including introns and exons. Genes with two polyadenylation sites that undergo APA with quiescence were enriched in genes involved in RNA splicing and processing (Table 2 and Additional file 9). Genes that undergo APA with quiescence also included genes involved in cell migration (Table 1).

### Reduced levels of mRNA processing factors in quiescent fibroblasts

To better understand the regulation of polyadenylation site use with quiescence, we monitored the levels of APA factors in proliferating and quiescent fibroblasts. Cleavage and polyadenylation of pre-mRNA transcripts are mediated by the coordinated activity of three core protein complexes [16]. The cleavage and polyadenylation specificity factor (CPSF) complex recognizes a hexameric sequence (AAUAAA or a similar sequence) in a 50-nt region upstream of the cleavage site [48, 49]; the 3′ pre-RNA, subunit 2, 64 kDa (CSTF2 or CstF-64) subunit of the CstF complex recognizes a U-rich or G/U-rich region about 20–40 nucleotides downstream of the cleavage site [19, 50–53]; and Nudix (nucleoside diphosphate linked moiety X)-type motif 21 (NUDT21 or CFIm25) recognizes UGUA sequences upstream of the cleavage and polyadenylation sites [54]. CPSF73, a component of the CPSF complex, is the endonuclease that performs the cleavage event at the hexameric sequence [55]. Increased levels of CSTF complex proteins have been associated with the use of proximal polyadenylation sites [19, 56, 57], while the CFIm complex has been reported to repress the use of proximal polyadenylation sites [45, 57, 58]. Our RNA-Seq data revealed that most of the core polyadenylation factors and auxiliary factors associated with cleavage and polyadenylation are modestly downregulated at the transcript level in quiescent compared with proliferating fibroblasts (Additional file 2). Among the core factors, CstF-64/CSTF2 is strongly and significantly (3.1-fold) downregulated at the transcript level. Using immunoblotting, we found that the protein levels of CstF-64, CPSF73, and CFIm25 are lower in 7dCI or 7dSS than in proliferating fibroblasts (Fig. 3d). By monitoring the extent of Serine 5 phosphorylation of RNA pol II carboxyterminal domain (CTD) as an indication of transcription initiation rate [59] with immunoblotting, we found that CstF-64 downregulation at the protein level with quiescence was stronger than the reduction in transcription initiation (Fig. 3d).

### Knockdown of cleavage and polyadenylation factors replicates polyadenylation site selection with quiescence

**Fig. 4:**
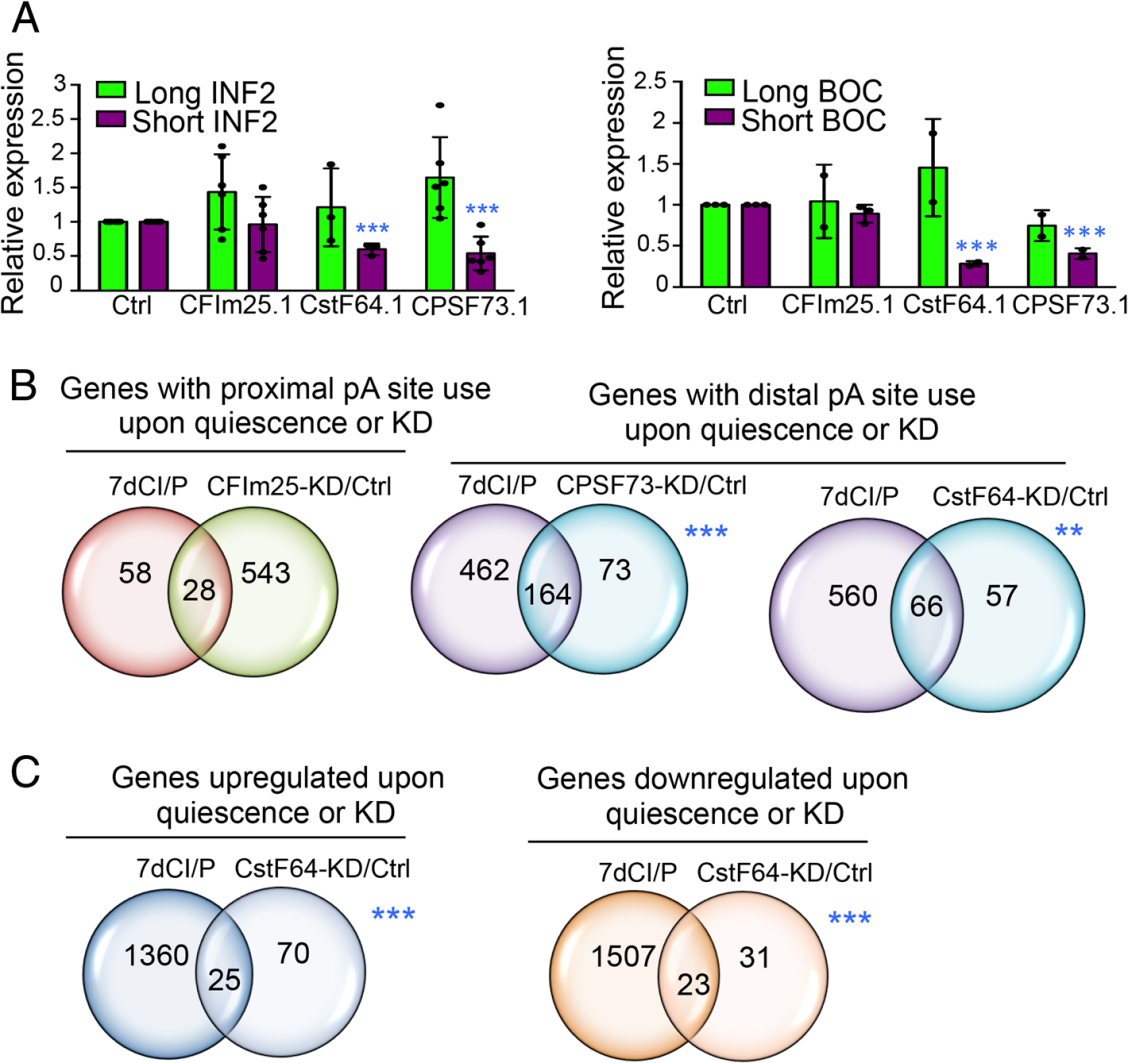
Knockdown of cleavage and polyadenylation factors results in changes in isoform use and gene expression that overlap with quiescence. **a** Knockdown of cleavage and polyadenylation factors induces a shift in isoform expression. Real-time PCR was performed for the short and long isoforms of INF2 and BOC in proliferating fibroblasts expressing a control siRNA or an siRNA that targets CFIm25, CstF-64, or CPSF73. The short isoform of INF2 or BOC was significantly reduced in cells transfected with an siRNA against CstF64 or CPSF73. Plots show individual datapoints as dots. Bar graphs represent mean and average ± S.D. The number of replicates for control, CFIm25 and CPSF73 knockdown for short and long INF2 is 6. The number of replicates for CstF64 knockdown for short and long INF2 is 3. The number of replicates for all conditions for long BOC is 2, except the control, which had 3 replicates. The number of replicates for control and CFIm25 knockdown for short BOC is 3. The number of replicates for CstF64 and CPSF73 knockdown for short BOC is 2. Statistical significance in knockdown cells compared to control cells was determined for long and short isoforms with two-tailed, unpaired t-tests. **b** Overlap among genes that undergo APA with quiescence and knockdown of cleavage and polyadenylation factors. The overlap between genes that use the proximal polyadenylation site with quiescence and use a proximal polyadenylation site preferentially with CFIm25 knockdown is shown on the left. Overlap between genes that use distal polyadenylation sites with quiescence and genes that use distal polyadenylation sites with CPSF73 or CstF64 knockdown are shown in the middle and the right, respectively. **c** Overlap between genes upregulated with quiescence and genes upregulated with CstF-64 knockdown (left) and overlap between genes downregulated with quiescence and genes downregulated with CstF-64 knockdown (right). The overlap between groups of genes was tested using the hypergeometric test

To better understand the role of cleavage and polyadenylation factors in polyadenylation site selection with quiescence, we introduced siRNAs that target CstF-64, CPSF73 or CFIm25, or a control siRNA, into fibroblasts. Strong knockdown of the targeted gene was confirmed with real-time PCR (Additional file 1: Figure S3). In comparison to control cells, knockdown of these polyadenylation factors did not significantly affect cell viability (Additional file 1: Figure S4A and B). We tested whether knocking down the expression of cleavage and polyadenylation factors results in changes in the levels of shorter and longer isoforms of genes that undergo APA with quiescence using real-time PCR primers designed to recognize the short or long isoforms of INF2 or BOC (Fig. 3a). For INF2, knockdown of CstF-64 or CPSF73, but not CFIm25, resulted in reduced levels of the short isoform of INF2 and an increase in the long isoform of INF2 (Fig. 4a). For BOC, knockdown of CstF-64 or CPSF73, but not CFIm25, resulted in lower levels of the short BOC isoform (Fig. 4a). Knockdown of CstF-64 resulted in an increase in the long isoform of BOC (Fig. 4a).

To monitor global APA changes, we performed polyadenylation site-enriched RNA-Seq of fibroblasts transfected with a control siRNA or an siRNA that targets a polyadenylation factor (CstF-64, CPSF73, or CFIm25) [47]. Knockdown in two different strains of fibroblasts resulted in highly reproducible results (Additional file 1: Figure S2B). Each knockdown resulted in significant changes (|RUD| > 0.05) in polyadenylation site selection, with CFIm25 knockdown resulting in a clear shift toward use of more proximal polyadenylation sites (Additional file 1: Figure S4C and Additional file 10), consistent with previous reports [60, 61]. We compared the genes that shift polyadenylation site use with quiescence with the results of knockdown of each cleavage and polyadenylation factor (Fig. 4b and Additional file 1: Figure S5A and B). Among the three polyadenylation factors, knockdown of CFIm25 resulted in the largest number of genes that shift to greater use of the proximal polyadenylation site (shorter isoforms), and the most genes that overlap with shifts to more proximal polyadenylation sites with quiescence (Fig. 4b and Additional file 1: Figure S5A). We observed significant overlap among the genes that use more distal polyadenylation sites (shift to longer isoforms) with quiescence and genes that use more distal polyadenylation sites with knockdown of each factor, with larger numbers of genes affected for CstF-64 or CPSF73 knockdown (Fig. 4b and Additional file 1: Figure S5A). Some of these changes in polyadenylation site use were specific for one factor, while some were regulated by more than one or even all three factors (Additional file 1: Figure S5B). For 626 unique genes that shift to distal polyadenylation site use with quiescence, 226 genes (36%) also shift to distal polyadenylation site use with knockdown of one or more polyadenylation factors. For 86 genes that shift to proximal polyadenylation site use with quiescence, 38 (44%) also shift to proximal polyadenylation site use with knockdown of one or more polyadenylation factors (Additional file 1: Figure S5B).

Knockdown of CstF-64 resulted in changes in gene expression that significantly overlap with gene expression changes with quiescence (Fig. 4c and Additional file 11). Gene expression changes upon knockdown of CPSF73 and CFIm25 overlapped with gene expression changes during quiescence as well, but fewer genes were involved (Additional file 1: Figure S5C).

Some of the genes that were regulated (APA changes or gene expression changes) with knockdown of CstF-64 was found to be associated with GO terms related to cell movement (Table 3). Several of these migration genes that undergo changes in APA upon CstF64 knockdown also did so with quiescence, such as Arp2/3 complex protein ACTR2 and CDC42 and RAC1-binding protein IQGAP1.

**Table 3:** List of genes involved in motility that are altered with CstF64 knockdown

Type of change (analysis)	Genes^a^
Gene expression (DESeq)	VIM, WDR1, ACTN1, *ACTB*, MAPK1, MYH9, BDKRB1, SEMA5A, FN1, IFT43, MYO1B, LMNA, HMGB2, GREM1, TPM4, DDIT4, IL8, TUBB4B, *TUBB*, PDCD6
Alternative polyadenylation (Relative usage of distal site)	*MAP4*, *SP100*, DYNC1I2, *TBX5*, OSBPL8, *RBFOX2*, BMPR1A, *LAMA4*, *DOCK7*, *RAP2C*, RHOT1, *LAMC1*, *ACTR2*, *IQGAP1*, RHOT2, *APP*, LMO4, *CERS2*, *GNA12*, EGFR, WASF2, *AMFR*, *SUN1*, *AMOTL1*, *STAT3*, BSG, *EXT1*, *COL18A1*, NTF3, SIAH1, *DPP4*, *EMP2*

^a^The genes that changed with quiescence for the same analysis are italicized

### Cleavage and polyadenylation factor recognition sites are more prevalent in genes that undergo alternative isoform use with quiescence

**Fig. 5:**
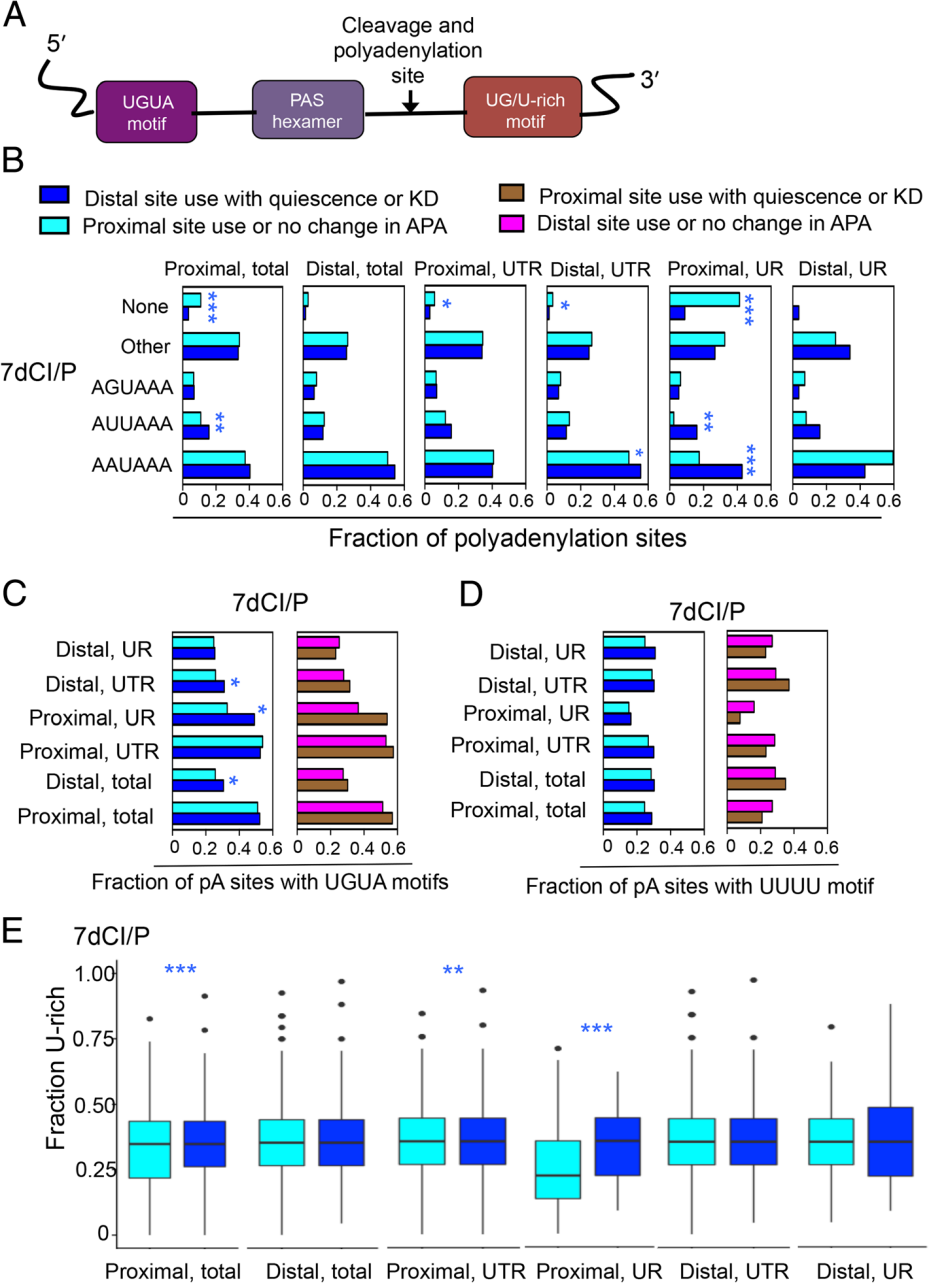
Changes in polyadenylation site recognition sequences in proximal versus distal polyadenylation sites for genes undergoing APA with quiescence. **a** Schematic showing the relative position of the UGUA motif, hexamers, the cleavage site and GU/U-rich motifs. **b** The frequencies with which different possible hexamers are present at the proximal or distal polyadenylation sites are shown for genes that have two polyadenylation sites and shift to the use of more distal polyadenylation sites with quiescence (dark blue). Other hexamers are AAACAU, AAUAAC, UUAAAG, UUAAAU, UAUAAA, AAUACA, CAUAAA, AAUAUA, GAUAAA, AAUGAA, AAGAAA, ACUAAA, AAUAGA, AAUAAU, AACAAA, AUUACA, AUUAUA, AACAAG, and AAUAAG. Data are compared with results for genes that use a proximal polyadenylation site or do not change their use of polyadenylation site with quiescence (light blue). Data are shown for all genes, for genes that undergo UTR APA and for genes that undergo UR APA. Statistically significant differences were determined by Fisher’s exact test (**c**) The fraction of genes with a UGUA motif in the region upstream of the polyadenylation site hexamer is shown. Data are provided for genes that shift to greater use of distal polyadenylation sites in quiescence (dark blue) and a control set of genes that do not use distal polyadenylaton sites more with quiescence (light blue) (left plots). Data are also provided for genes that shift to greater use of proximal polyadenylation sites with quiescence (brown) and a control set of genes that do not shift to greater use of proximal polyadenylation sites (pink) (right plots) Statistically significant differences were determined by two-tailed Fisher’s exact test. **d** The fraction of genes with a U-rich motif in the region downstream of the polyadenylation site hexamer are shown. **e** The fraction of base pairs 20–40 nts downstream from the polyadenylation site that are Us is shown for genes that shift to use of more distal polyadenylation sites with quiescence. Statistical significance was determined by Wilcoxon signed-rank test

To further understand the importance of different cleavage and polyadenylation site factors in the alternative use of polyadenylation sites with quiescence, we monitored the presence of their recognition motifs (Fig. 5a). For genes that undergo UR APA and shift to greater use of more distal polyadenylation sites during quiescence, their proximal polyadenylation site is more likely to have a strong hexamer (AAUAAA or AUUAAA), and less likely to have no hexamer, than for control genes (Fig. 5b). Similarly, when CPSF73 is knocked down, genes that shift to greater use of distal polyadenylation sites are less likely to have no hexamer than genes that do not lengthen with quiescence (Additional file 1: Figure S6). The findings support a role for reduced CPSF73 levels contributing to the use of more distal polyadenylation sites in genes undergoing UR APA in quiescent cells.

Extending the analysis to UGUA motifs recognized by CFIm25, among genes that use UR APA to shift to more distal polyadenylation site use in quiescent than proliferating cells, there was a significantly higher chance of a UGUA motif being present at the proximal site than for a control set of genes (Fig. 5c). With CFIm25 knockdown, the strongest effect was increased use of proximal polyadenylation sites, and the genes affected were more likely to have a UGUA motif at their distal polyadenylation site (Additional file 1: Figure S7).

To monitor the presence of binding sites for CstF-64, we determined the fraction of polyadenylation sites that contain a string of four or more uracils in the region 20–40 base pairs downstream of the polaydenylation site. With this analysis, there were more UUUU motifs at proximal polyadenylation sites among genes that shift to the use of more distal sites with quiescence, but the difference was not statistically significant (0.098) (Fig. 5d). We also monitored the fraction of U’s (U-rich) and the fraction of U’s or G’s (UG-rich) in the same 20–40 base pair region. Proximal polyadenylation sites were enriched in U-rich and UG-rich sequences for genes that shifted to greater use of longer isoforms with quiescence (Fig. 5e and Additional file 1: Figure S8). This result is consistent with downregulation of CstF-64 playing a role in the shift to more distal polyadenylation sites with quiescence. Thus, in proliferating conditions, CstF-64 levels are more available for binding to U-rich proximal sites, which supports the generation of shorter isoforms.

### Shifting to more distal polyadenylation sites stabilizes transcripts in quiescent but not proliferating fibroblasts

**Fig. 6:**
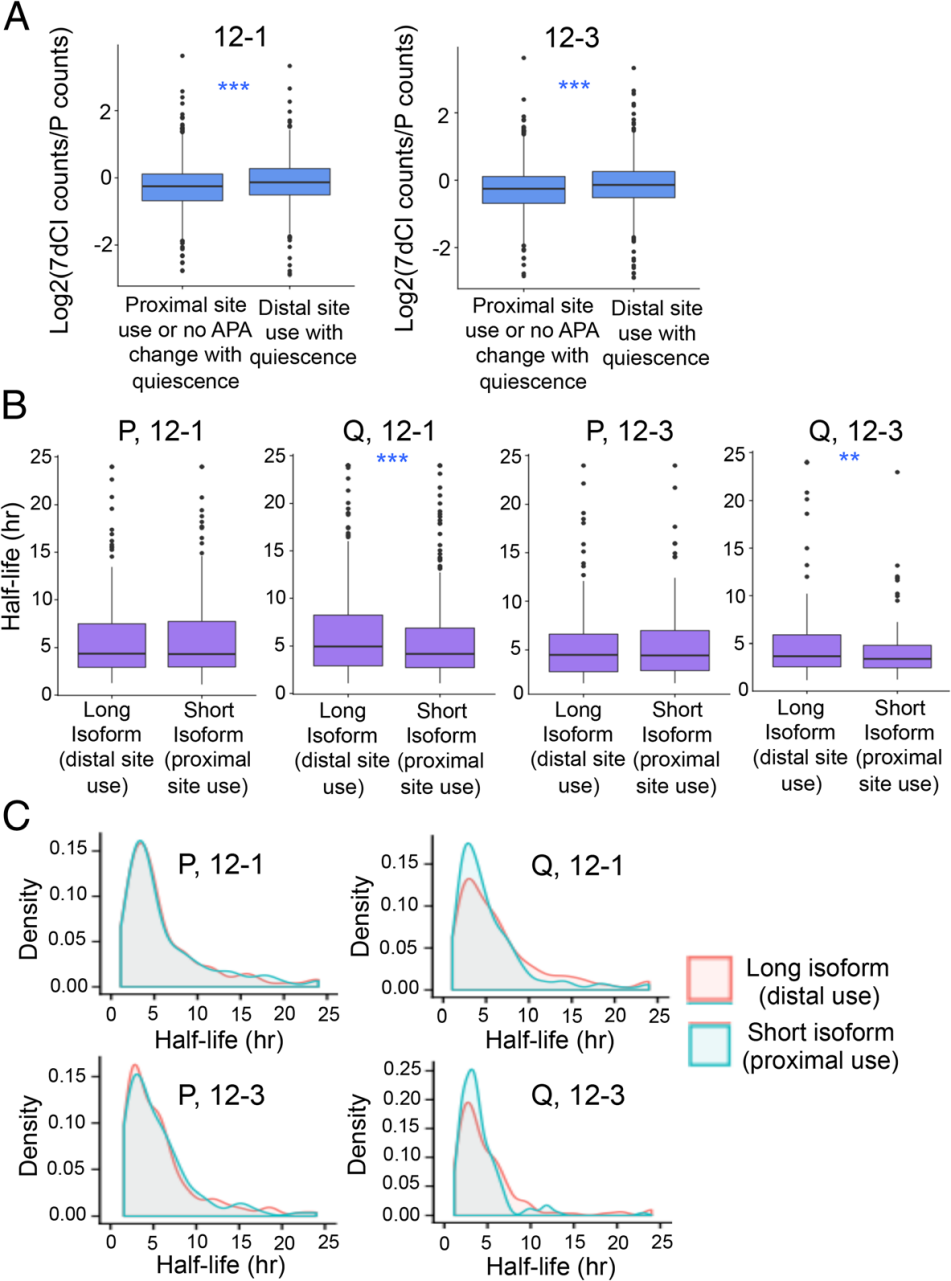
Higher expression and increased stability for genes that shift to greater reliance on distal polyadenylation sites in quiescence. **a** For two different fibroblast lines (12–1 and 12–3), the log_2_(7dCI counts/Proliferating counts) is plotted for genes that shift to increased use of more distal polyadenylation sites with quiescence and a control group that does not shift to more distal site use. Boxes indicate 25 to 75% ranges and whiskers indicate minimum and maximum values. Statistical significance was determined with Wilcoxon signed-rank test. The ratio of expression level in 7dCI versus P was higher for genes that shift to more distal polyadenylation site use with quiescence for both 12–1 and 12–3 fibroblasts. **b** Isoform-specific transcript decay half-lives were determined for 12–1 and 12–3 strains of fibroblasts in proliferating and quiescent conditions. Box plots show the range of half-lives for isoforms that terminate at proximal polyadenylation sites and isoforms that terminate at more distal polyadenylation sites in proliferating and quiescent conditions. Long isoforms are significantly more stable in quiescent but not proliferating states in 12–1 and 12–3 fibroblasts. Statistically significant differences were determined by Wilcoxon signed-rank test. **c** Density plots of half-lives for isoforms that terminate at proximal or distal polyadenylation sites in proliferating and quiescent fibroblasts from strains 12–1 and 12–3

Changes in the levels of transcripts that terminate at different polyadenylation sites could reflect changes in the rates that these isoforms are generated based on the levels of polyadenylation factors, or changes in the rates at which they decay. To understand the relationship between polyadenylation site selection and transcript fate, we first determined whether APA with quiescence was associated with a change in gene expression. Relative expression in quiescent compared with proliferating fibroblasts was slightly higher on average for genes that undergo a shift to greater use of distal polyadenylation sites with quiescence than for genes that do not undergo APA or use the proximal polyadenylation site preferentially in quiescence (Fig. 6a, *p* < 0.001, Wilcoxon signed-rank test). This finding would be consistent with longer transcripts being more stable.

To better understand the relationship between polyadenylation site selection and transcript decay rate, we added actinomycin D to inhibit new transcription in proliferating or 7dCI fibroblasts, collected RNA over a timecourse, and performed polyadenylation site-enriched RNA-Seq to monitor the rate that different gene isoforms decayed [62]. The results extend our previous studies of genome-wide transcript decay rates in proliferating and 7dCI fibroblasts using microarrays [63]. In two different fibroblast strains (12–1 and 12–3), we found that isoforms terminating at distal polyadenylation sites were more stable than isoforms terminating at proximal polyadenylation sites in quiescent, but not proliferating, fibroblasts (Additional file 12 and Fig. 6b, c).

We identified motifs enriched in the interpolyadenylation site regions in genes that shift to a longer isoform with quiescence. Among the RNA-binding proteins that bind to these motifs, some are induced in quiescent compared with proliferating cells and would be candidates for stabilizing longer transcripts in quiescent cells (Additional file 1: Table S5). Our findings indicate that the shift to the use of longer isoforms in quiescent cells results in an overall stabilization of transcripts and a modest increase in expression levels. Therefore, the higher levels of longer isoforms in quiescent than proliferating fibroblasts could reflect both a difference in polyadenylation site selection (influenced by levels of polyadenylation factors) and a difference in the rate at which the shorter and longer transcripts decay in the two proliferative states.

### Cleavage and polyadenylation factors are expressed at higher levels in wound-healing than quiescent skin in vivo

**Fig. 7:**
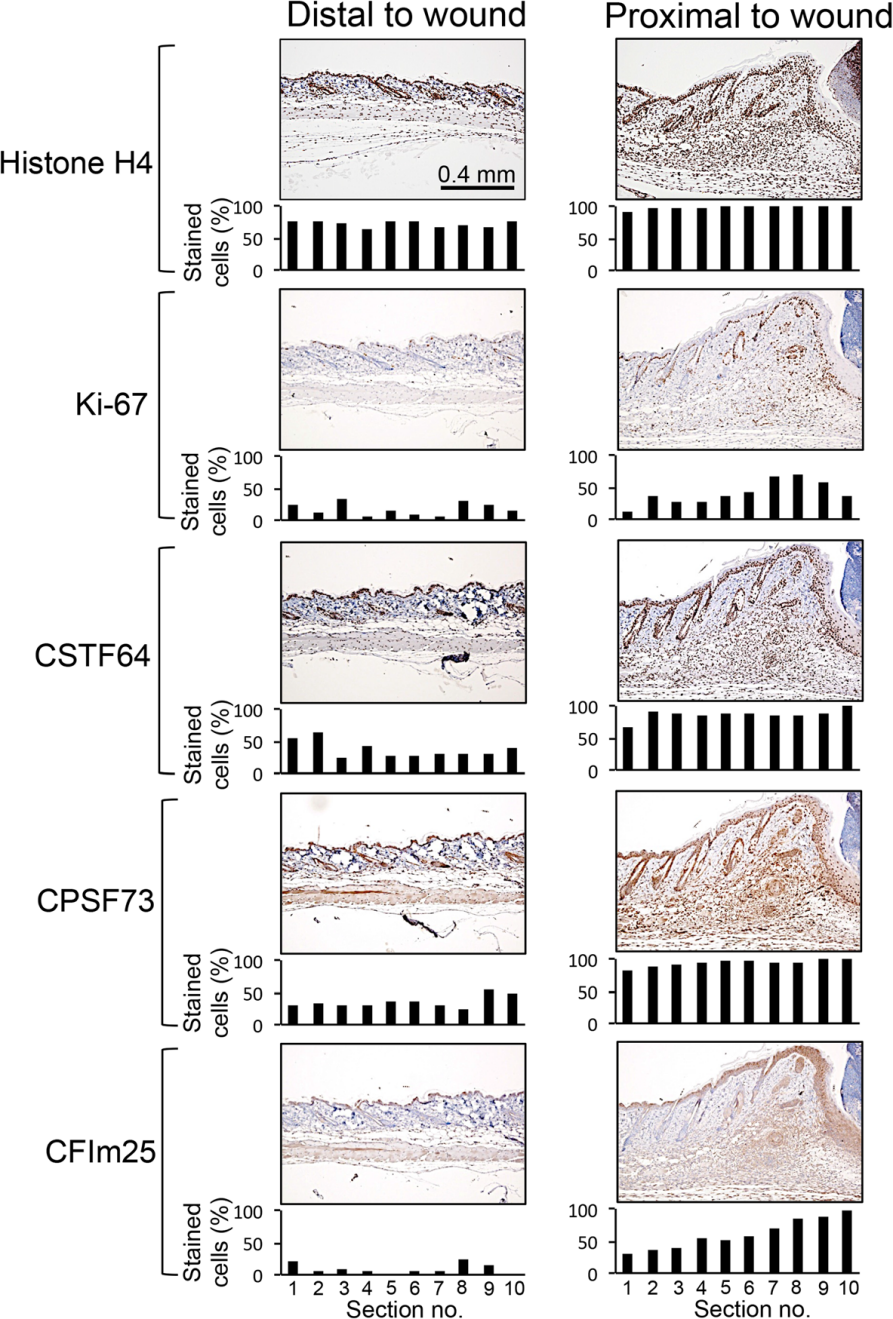
Cleavage and polyadenylation factors are expressed at higher levels in fibroblasts near a wound than in fibroblasts of healthy skin. Mouse skin was collected 5 days after introduction of a punch biopsy. Normal mouse skin was collected 2 cm away from the wound. Samples were stained with immunohistochemistry for proliferation marker Ki-67, histone H4 as a control, or alternative polyadenylation and cleavage factors CstF-64, CPSF73 or CFIm25 (brown). Samples analyzed with immunohistochemistry were counterstained with hematoxylin (blue nuclei). Individual cells at different positions from the wounds were assigned positive or negative staining and the percentages are shown. Ki-67 does not label all dividing cells, and likely underestimates the fraction of cells that are actively cycling [122]. Levels of all three cleavage and polyadenylation factors were higher in the fibroblasts, myofibroblasts and immune cells proximal to a wound than in the fibroblast-rich dermal areas of healthy skin distal to the wound

Wound healing is a situation in which cells are activated to both proliferate and migrate. We investigated the levels of cleavage and polyadenylation factors in normal skin and in dermal excisional wounds in mice. We introduced punch biopsies into the backs of mice and collected wounded tissue and unwounded control skin approximately 2 cm from the wound. Immunohistochemistry for the proliferation marker Ki-67 revealed higher levels of proliferation of a migrating mass of cells that includes fibroblasts, myofibroblasts, and immune cells in the skin proximal to the wound compared with cells in the dermis of control, unwounded skin (Fig. 7) [64]. Immunostaining for histone H4 as a control revealed similar staining in wounded and control skin as expected. Immunohistochemistry for CstF-64, CPSF73, or CFIm25 revealed a higher fraction of cells with positive nuclei in the region surrounding the wounded skin for all three factors than in control, unwounded skin (Fig. 7). This analysis revealed that the shift toward higher levels of cleavage and polyadenylation factors in proliferating fibroblasts in culture also occurs in the migratory, proliferating cells that heal wounds in vivo.

### CstF-64 knockdown reduces fibroblast migration

**Fig. 8:**
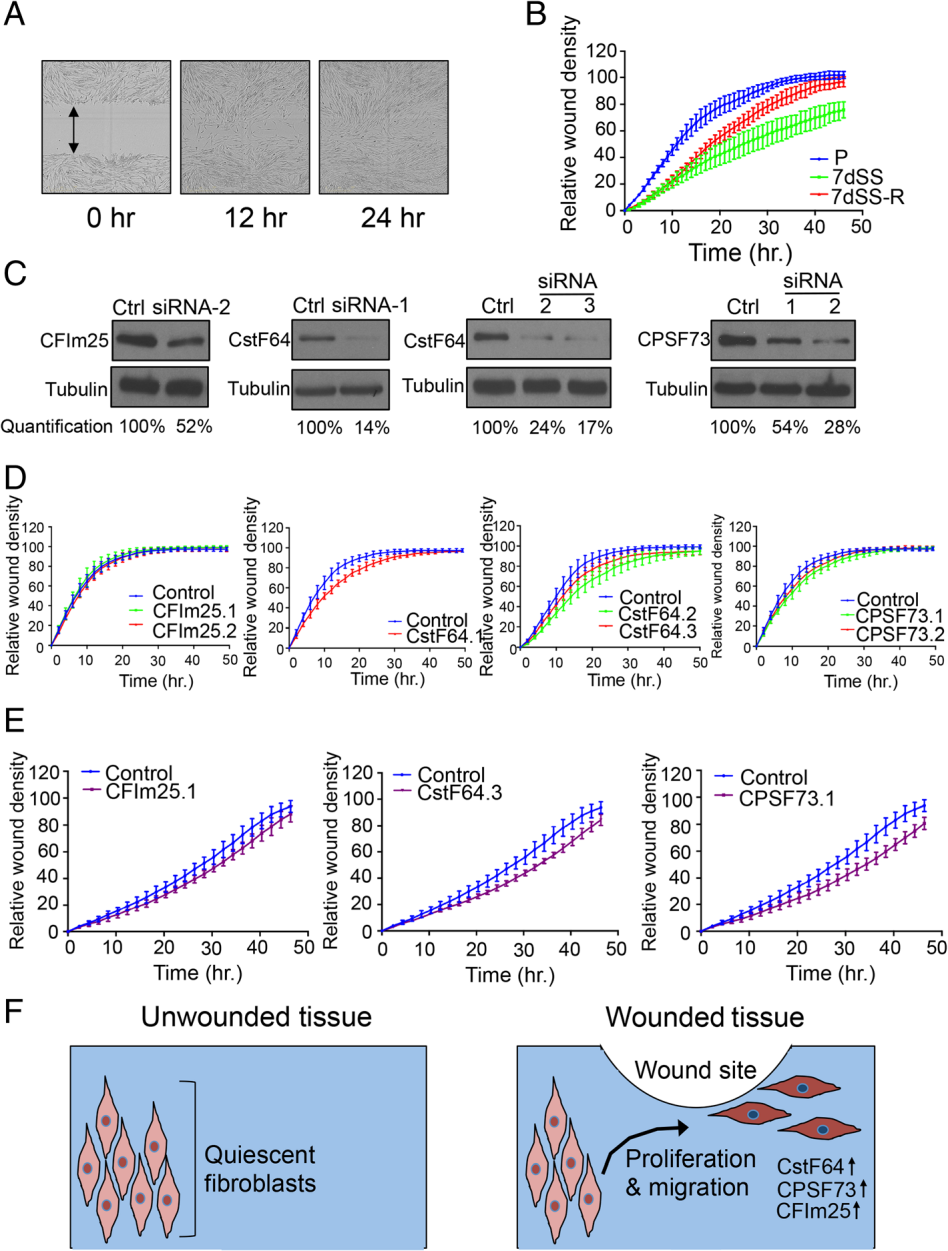
Knockdown of APA factors results in reduced migration. **a** Example of Incucyte migration assay. Bright-field images from an assay monitoring the rate of migration into a denuded area (marked by a double-arrow) performed with Incucyte real-time imaging are shown. **b** Proliferating fibroblasts migrate more rapidly into a denuded area than quiescent fibroblasts. Fibroblasts were sampled in proliferating conditions, 7dSS conditions (7dSS), or after 7dSS followed by serum restimulation (7dSS-R). Fibroblasts were plated into 96-well plates and a portion of the well was denuded of cells. Plates were analyzed with an Incucyte real-time imaging instrument and the associated software to monitor the rate at which fibroblasts migrated into the denuded area. The ratio of cell density in the denunded area to the non-denuded area (relative wound density) over a time-course is plotted. Six wells were monitored for each condition and data represent mean and standard deviation. Proliferating versus 7dSS samples (*p* value < 0.001, repeated measures two-way ANOVA with Dunnett’s multiple comparison test), proliferating versus 7dSS-restimulated samples (*p* value < 0.001), and 7dSS versus 7dSS-R (*p* value < 0.001) were statistically significantly different. **c** Immunoblots demonstrating knockdown of the targeted cleavage and polyadenylation factor by siRNAs in fibroblasts. The percent knockdown of protein level is also shown. **d** Knockdown of CstF-64 reduces fibroblast migration. Fibroblasts were transfected with a control siRNA or an siRNA against CFIm25, CstF-64, or CPSF73. CstF-64-knockdown fibroblasts exhibited reduced migration into a denuded area than control fibroblasts (CstF64.1 *p* value = 0.0013). Two additional siRNAs against CstF-64 (CstF64.2 and CstF64.3) reduced migration compared with a matched control siRNA as well (CstF64.2 *p* value = 0.0021, CstF-64.3 *p* value = 0.0384). Six replicates were performed for each condition. **e** Knockdown of CstF-64 or CPSF73 reduced migration of triple negative breast cancer cells. Triple negative breast cancer cell line MDA-MB-231 was transfected with a control siRNA or an siRNA against CstF-64, CPSF73 or CFIm25. Migration into a denuded area on the plate was monitored with an Incucyte instrument. Knockdown of CstF-64 or CPSF73 resulted in reduced migration (CstF64 *p* value = 0.0002, CPSF73 *p* value = 0.0013). For all conditions, the number of replicates for each condition was 6. **f** Schematic diagram showing elevated cleavage and polyadenylation factors in fibroblasts in the wound-healing environment. Increased expression of CstF-64, CPSF73, and CFIm25 in fibroblasts in wounds is expected to result in increased use of proximal polyadenylation sites and may promote fibroblast migration to the wound

Based on the consistency with which we observed changes in the mRNA processing and expression of genes important for cell motility in proliferating versus quiescent fibroblasts (Table 1), we hypothesized that changes in mRNA processing associated with the transition between proliferation and quiescence are also important for the closely linked process of cell migration. First we tested the association between proliferation and migration. We generated fibroblasts that were proliferating, induced into quiescence by 7dSS, or restimulated after 7dSS by re-addition of medium with serum. We monitored the rate at which fibroblasts in each condition migrated into a denuded area on a tissue culture plate with real-time imaging (Fig. 8a). Migration was quantified as the ratio of cell concentration in the denuded area compared to the cell concentration in the non-denuded area, thus normalizing for possible differences in proliferation rate. We discovered that the proliferating and restimulated fibroblasts migrated into the denuded area more rapidly than the serum-starved fibroblasts (Fig. 8b).

We observed changes in the transcript and protein levels of cleavage and polyadenylation factors as fibroblasts transition between proliferation and quiescence. To test whether levels of cleavage and polyadenylation factors change in fibroblasts induced to migrate into a denuded area, we introduced denuded areas into cultures of fibroblasts and performed immunofluorescence to monitor the levels of cleavage and polyadenylation factors. CstF-64 and CPSF73 levels were significantly higher in the cells that had migrated into the denuded area than cells that had not migrated, while no significant change was observed for CFIm25 (Additional file 1: Figure S9). We then tested the importance of alternative polyadenylation factors for fibroblast motility. We generated knockdown fibroblasts with control siRNAs or siRNAs against cleavage and polyadenylation factors, and monitored the rate of migration. Knockdown of CstF-64 with any of three different siRNAs (Fig. 8c) resulted in reduced migration into the denuded area (Fig. 8d). CstF-64 siRNA #1 had the strongest effect on CstF-64 levels and resulted in the most significant reduction in migration. Knockdown of CPSF73 (Fig. 8c) resulted in slower migration, but the difference was not statistically significant (Fig. 8d). Knockdown of CFIm25 (Fig. 8c) did not affect migration rate (Fig. 8d). Thus, CstF-64 is induced in migrating cells, and knockdown of CstF-64 resulted in APA changes and downregulation of genes that overlap with those that occur with quiescence, including genes associated with cell migration (Table 3). These findings are consistent with our observation here that knockdown of CstF-64 simulates the reduced migration observed for quiescent fibroblasts.

### Knockdown of cleavage and polyadenylation factors reduces migration of triple negative breast cancer cells

To determine the generality of our findings for different types of cells, we tested the effects of siRNAs targeting CstF-64, CPSF73 or CFIm25 on the migration of triple negative breast cancer cells (Additional file 1: Figure S3). Triple negative breast cancer is a highly aggressive breast cancer subtype characterized by a lack of hormonal receptors and an absence of HER2 amplification [65]. Knockdown of CstF-64 or CPSF73 resulted in significantly reduced migration of triple negative breast cancer cells (Fig. 8e). The triple negative breast cancer cells were even more sensitive to altered polyadenylation site selection than the fibroblasts, which may reflect the increased reliance of cancer cells on proximal polyadenylation sites [20, 45, 46, 66]. Our results demonstrate that the selection of polyadenylation sites can affect the migratory capacity of cancer cells as well as fibroblasts in wound healing (Fig. 8f).

## Discussion

While we and others have shown that the transition to quiescence is associated with widespread changes in gene expression [9–11], and others have previously shown changes in the selection of polyadenylation sites with quiescence [21], we sought here to better understand the relationship between quiescence and alternative polyadenylation. Gene expression analysis of RNA-Seq data revealed that genes involved in mRNA processing (splicing and polyadenylation) are downregulated as fibroblasts enter quiescence (Fig. 1c, d). These findings suggested to us that processing of pre-mRNA transcripts may be different in quiescent compared with proliferating cells, and that these changes may contribute to changes in transcript abundance and the functional attributes of proliferating versus quiescent fibroblasts. We further discovered through differential exon analysis of RNA-Seq data that hundreds of genes exhibit changes in isoform expression during the transition to quiescence. Quiescent fibroblasts expressed lower levels of some auxiliary splicing factors (Fig. 2b) and were more likely to include exons and retain introns than proliferating fibroblasts (Fig. 2a), demonstrating cell-cycle state-dependent changes in splicing and intron retention [38]. Introns that were retained tended to have splicing motifs that varied from the consensus sequence, especially for the polypyrimidine tract adjacent to 3′ splice sites in the proliferating state (Fig. 2c), potentially reducing the effectiveness of splicing factors or associated RNA binding proteins. Our results are consistent with a model in which quiescence is associated not with a complete shut-down of mRNA processing events, but rather with a shift in the processing of specific transcripts such that, in addition to changes in gene expression, an alternative set of exons and isoforms are present in fibroblasts that are proliferating versus quiescent. Genes involved in cell motility were among those demonstrating consistent changes in splicing in proliferating versus quiescent cells (Table 1).

Among the changes in isoform use that we observed, the most prominent effect was a change in the selection of polyadenylation sites in proliferating versus quiescent fibroblasts. In response to quiescence induced by contact inhibition, 714 genes exhibited a change in polyadenylation site selection, and in 88% of instances, alternative polyadenylation site use resulted in a lengthening of transcripts in quiescent compared with proliferating cells (Fig. 3c). These findings are consistent with previous studies that revealed that 3′ UTRs are shorter in more rapidly proliferating cells [18, 21], stem cells [67], and cells and tissues derived from tumors [20, 46, 68], and longer in cells that divide less frequently such as differentiated tissues [13, 15, 67]. We found that 3′ UTR lengthening reverses when quiescent cells re-enter the cell cycle (Fig. 3b), demonstrating that these changes can be reversed based on proliferative state.

To better understand the basis for the changes in polyadenylation site selection in proliferating versus quiescent fibroblasts, we monitored the levels of polyadenylation factors in proliferating and quiescent cells. Transition to quiescence was associated with lower levels of cleavage and polyadenylation factors CstF-64, CFIm25, and CPSF73 (Fig. 3d). Knockdown of each these three factors resulted in changes in polyadenylation site use that overlapped significantly with the changes that occurred with quiescence (Fig. 4b and Additional file 1: Figure S5A and B). There were also changes in gene expression as a result of knockdown of specific factors, especially CstF-64. These gene expression changes overlapped with changes in gene expression that occur with quiescence (Fig. 4c and Additional file 1: Figure S5C).

To further understand the contribution of different cleavage and polyadenylation complexes to the shift in polyadenylation site selection with quiescence, we monitored the presence of their recognition sites. For genes that use more distal upstream region polyadenylation sites with quiescence, the proximal hexamer was much more likely to match the canonical hexamer, and very unlikely to be absent (Fig. 5b). A similar shift was observed with CPSF73 knockdown (Additional file 1: Figure S6A). This is consistent with reduced expression of CPSF73, and reduced use of upstream region proximal polyadenylation sites, as a factor contributing to the lengthening of transcripts with quiescence. A role for reduced CstF-64 levels in quiescent cells promoting the shift to more distal polyadenylation sites is supported by the finding that the sequence between 20 and 40 bps downstream of the proximal polyadenylation site included more Us on average and more Gs and Us on average, for genes that use more distal polyadenylation sites with quiescence (Fig. 5e). Taken together, the results support the importance of reduced levels of cleavage and polyadenylation factors with quiescence, with the polyadenylation pattern for specific sequences determined in part by the presence or absence of binding factors for the reduced factors.

Some previous studies have reported that shorter transcripts generated by alternative polyadenylation tend to be expressed at higher levels than the corresponding longer isoform [20, 46, 69, 70], while other studies have found little effect of alternative polyadenylation on transcript levels, transcript stability or protein abundance [71, 72]. Additional studies have found that shorter transcripts can be more or less stable [71, 73], and two detailed analyses in yeast showed clear examples of stability elements in 3′ UTRs that make longer isoforms more stable than shorter isoforms [74, 75]. In our study, we observed that genes with longer 3′ UTRs during quiescence, on average, exhibited a small but significant increase in expression level during quiescence compared to proliferating cells (Fig. 6a). Further, isoforms are more stable when distal rather than proximal polyadenylation sites are used in the quiescent state, but decay rates are similar when proximal or distal sites are used in the proliferating state (Fig. 6b, c). The findings are consistent with induction of an RNA-binding proteins in quiescent cells that bind to motifs present in the region between the polyadenylation sites and limit transcript degradation when the cells are quiescent. There are multiple motifs recognized by RNA-binding proteins in this inter-polyadenylation site region, and some of the factors that recognize these motifs are expressed at higher levels in quiescent than proliferating fibroblasts (Additional file 3). The findings are also consistent with the retention of longer transcripts in ribonucleoprotein storage granules or other structures in quiescent cells [76]. These changes could contribute to the higher gene expression levels of transcripts undergoing transcript lengthening in quiescence (Fig. 6a).

In many [20], but not all [77], studies, cancerous tissue and cancer cell lines were found to be more likely to express transcripts that terminate at proximal than distal polyadenylation sites, consistent with our observations in proliferating fibroblasts. Different polyadenylation factors have been found to have distinct effects on APA. Downregulation of CFIm25 repressed proximal polyadenylation site use (Additional file 1: Figure S4C) consistent with previous reports [45, 54]. Depletion of CFIm25 has been found to enhance the tumorigenic properties of glioblastoma cells [45], while overexpression of CFIm25 reduced tumor growth [45]. Shortening of 3′ UTRs has been associated with poor prognosis in breast and lung cancer [78]. Further, in an analysis of multiple tumor datasets deposited in The Cancer Genome Atlas, expression of CstF-64 correlated most closely with shortening of transcripts, with CPSF73 showing the next best correlation among the factors investigated [46]. Expression of shorter 3′ UTRs was an important predictor of patient outcome even beyond established clinical attributes [46]. In another study, CstF-64 expression was found to be associated with poor prognosis in lung cancer and its overexpression increased lung cancer cell proliferation and invasion [79]. In our dataset, cyclin D1 was the most strongly downregulated gene when CstF-64 was knocked down (Additional file 11), raising the possibility that CstF-64 levels modulate polyadenylation site selection and cyclin levels. Taken together with our data demonstrating that downregulation of CstF-64 in triple negative breast cancer cells reduces their migration (Fig. 8e), the data as a whole suggest that CstF-64-mediated APA may play an important role in regulating polyadenylation site selection, gene expression, cancer cell migration, metastasis, and patient outcome.

Fibroblasts transition from quiescence to proliferation and become more migratory in the context of wound healing. Some previous studies have supported a role for mRNA processing in wound healing [80–83]. By investigating the wound healing response in mice, we found that the levels of polyadenylation factors CstF-64, CFIm25, and CPSF73 were significantly higher in the area adjacent to the wound than distal to the wound (Fig. 7), similar to our finding that these factors are expressed at higher levels in proliferating than quiescent fibroblasts in culture (Fig. 3d). The results support a possible role for alternative polyadenylation in the proliferative and migratory changes that occur in the wound healing process.

Previous studies have identified mechanistic links between fibroblast proliferation and migration. Mitogen binding to receptor tyrosine kinases can activate focal adhesion kinase (FAK) and thereby stabilize focal adhesions [84, 85]. Activation of receptor tyrosine kinases can also recruit WASp [86], which promotes the formation of branched actin filaments that promote cell migration. The anti-proliferative cyclin-dependent kinase inhibitor p27^Kip1^ binds to and inhibits the activity of RhoA GTPase [87], an important regulator of actin dynamics and adhesion, spreading and migration [88]. Our findings that downregulation of APA factors, as occurs in response to antiproliferative signals via E2F transcription factors [21], reduces the capacity of fibroblasts to migrate into a denuded area, represents another mechanism linking fibroblast proliferation to migration through APA. We found that CstF-64 is induced in migrating cells, and knockdown of CstF-64 resulted in changes in polyadenylation site selection, altered expression of several migration genes (Table 3), and reduced cell migration (Fig. 8d). Among the genes expressed at lower levels with CstF-64 knockdown are beta actin, α-actinin, and myosin 1b. Our findings support a model in which changes in the selection of polyadenylation sites or changes in gene expression mediated by the levels of alternative polyadenylation factors play an important role in critical cell functions including migration. In a separate manuscript, we investigate in more detail the effects of isoform changes in one particular gene, RECK (included in Table 1 under UR-APA), on migration [89]. Taken together, our data and the data emerging from other laboratories, underscore the importance of CstF-64 as an important regulator of cellular functions, including migration, in multiple cellular contexts.

## Conclusions

Our work demonstrates that, in addition to changes in gene expression, the shift from a proliferating to a quiescent state is associated with changes in intron and exon inclusion and with the selection of polyadenylation sites. Overall, quiescent cells tend to retain introns and express longer transcripts that are present at higher levels and are more stable. Cleavage and polyadenylation factor CstF-64 is more abundant in proliferating fibroblasts in culture and in fibroblasts near a denuded area or a wound in mice. Knockdown of CstF-64 recapitulates changes in isoform use and gene expression in quiescent cells, and results in reduced cell migration in fibroblasts and cancer cells. Fibroblasts are often induced to proliferate and migrate in similar situations, and our data indicate that changes in the levels of CstF-64 can serve as a link between proliferative cues and migratory capacity.

## Methods

### Cell culture

Human foreskin fibroblasts were isolated from human skin obtained from the National Disease Research Interchange (NDRI) as described previously [24, 90]. Cells were seeded at 5 × 10^5^ cells per 10 cm dish for each cell cycle state and grown in Dulbecco’s modified Eagle medium (DMEM) (Life Technologies, Grand Island, NY) supplemented with 10% fetal bovine serum (FBS) (Atlanta Biologicals, Flowery Branch, GA and Corning, Thermo Fisher Scientific, Waltham, MA) at 37 °C in a 5% CO_2_ incubator. Detailed procedures for culturing proliferating and quiescent fibroblasts are described in [91]. Briefly, proliferating fibroblasts were collected for analysis 2 days after plating (60–80% confluent). 7dCI fibroblasts were collected 7 days after plating, or at an equivalent density, while 7dSS fibroblasts were seeded in full serum medium (10% FBS in DMEM), changed to reduced serum medium (0.1% FBS in DMEM), and collected 7 days after adding the reduced serum medium. Medium was changed every 2 days for both 7dCI and 7dSS fibroblasts. Restimulated samples were prepared by first performing the relevant quiescence arrest and readding the limiting factor. Restimulated fibroblasts were monitored with Incucyte migration assays or collected 24 or 48 h later for real-time PCR analysis. The triple negative breast cancer cell line MDA-MB-231 cell line (generous gift of the Banerjee and Christofk laboratories) was grown in 10% FBS in DMEM.

### RNA isolation for RNA-Seq and microarray analysis

RNA-Seq was performed on three biological replicates of fibroblasts isolated from two different donors, 12–1 and 10–5. Medium was aspirated from tissue culture plates of fibroblasts, and the attached cells were washed with 5 ml of PBS. Attached fibroblasts were lysed into 1 mL of Trizol reagent (Life Technologies, Carlsbad, CA) per 10 cm plate for 5 min. RNA was isolated from Trizol lysates as previously described [92, 93]. RNA concentrations were determined using a Nanodrop Spectrophotometer (Thermo Fisher Scientific Inc., Waltham, MA). RNA quality was verified on a Bioanalyzer 2100 (Agilent Technologies, Santa Clara, CA) using reagents from the RNA Nano 6000 kit (Agilent Technologies).

### RNA-Seq analysis

cDNA libraries were constructed using the Illumina TruSeq mRNA sample preparation kit (Illumina Inc., San Diego, CA) according to the manufacturer’s instructions for revision A of the protocol (Illumina Part #15008136). The low-input protocol was followed for all samples, and 1 to 10 μg of total RNA input was used per library (unstranded). Single-end 140 bp reads were generated on an Illumina HiSeq 2000 Instrument. Reads with Illumina (PHRED-based) quality scores above 10 (90% accuracy) were mapped to the hg19/GRCh37 build of the human genome using the TopHat (version 2.0.9) genome alignment algorithm [94, 95]. The bowtie indices for human were obtained from the bowtie website: http://bowtie-bio.sourceforge.net/tutorial.shtml. The standard workflow for Tophat alignment was followed as described here: https://ccb.jhu.edu/software/tophat/manual.shtml.

The default parameters for alignment as described in the Tophat manual were used. Standard DESeq (version 1.22.0) workflow [96] (https://bioconductor.org/packages/release/bioc/html/DESeq.html) was used to convert the output of TopHat (BAM files) to a file format with gene identifiers (UCSC gene annotation, GRCh37/hg19 assembly, date of access June, 2013) and read counts normalized for sequencing depth across the different biological samples and cell cycle conditions. Information about biological replicates was provided as input for variance calculations to determine differential expression among proliferating and 7dCI conditions in DESeq. To identify differentially expressed genes, the log_2_ (7dCI read count/proliferating read count) was used to compare expression differences between the two states. Genes with differences in read counts between conditions (proliferation versus 7dCI), and low variance in expression within the three biological replicates of each condition, were called significant by DESeq after multiple hypothesis correction (FDR < 5%) [97]. Heat maps were generated using the heatmap2 function of gplots package (2.12.1) (https://cran.r-project.org/web/packages/gplots/index.html) implemented in the R programming language [98, 99].

### Gene set enrichment analysis

For RNA-Seq data, gene sets with significantly different expression between proliferating and quiescent fibroblasts were identified using a Wilcoxon rank-sum test comparing the log fold-change estimates of genes within each set to genes not within the set [100]. Graphics were created using the GSEMA package implemented in R [101].

### Differential isoform analysis

To determine differential isoform use between proliferating and quiescent fibroblasts, the standard DEXSeq (version 1.14.2) workflow (https://bioconductor.org/packages/release/bioc/html/DEXSeq.html) [29] was followed. BAM files generated by aligning RNA-Seq reads to the human genome (hg19/GRCh37 build) were converted to gene-normalized read count files using exons as the identifiers. The Ensembl gene annotation (GRCh37 assembly) file was obtained from https://ccb.jhu.edu/software/tophat/igenomes.shtml. Differential exon expression was determined across the three biological replicates. Genes with significant differences in expression for specific exons (adjusted *p* value < 0.05) between proliferating and 7dCI conditions were used for further analysis.

### Microarray gene expression analysis

An aliquot of the same total RNA that was analyzed by RNA-Seq was also analyzed by microarray. Total RNA was reverse-transcribed into cDNA and fluorescently labeled with Cyanine 3-CTP (7dCI samples) or Cyanine 5-CTP (proliferating samples) with the Quick Amp Labeling Kit for Microarray Analysis (Agilent Technologies, Santa Clara, CA) following the manufacturer’s protocol. cRNA samples that passed yield and labeling standards were fragmented, and proliferating and quiescent samples were hybridized to two-color Human gene expression 4 × 44 K microarrays (Agilent Technologies) for 17 h at 65 °C in an oven rotating the arrays at 10 rotations per minute. Fluorescence intensities were detected using the Genepix scanner (Agilent Technologies) and probe identities were determined using Agilent’s feature extractor version 11.5. Probes detected over background fluorescence thresholds were used in subsequent gene expression analyses to calculate log_2_ (7dCI_intensity_/P_intensity_).

### Differential splicing analysis

RNA-Seq reads (fastq files) from three replicates of proliferating fibroblasts and three replicates of 7dCI fibroblasts were analyzed with the rMATS algorithm release 3.2.1 (http://rnaseq-mats.sourceforge.net/rmats3.2.1.beta/) [31–33] using Ensembl gene annotation (GRCh37 assembly). Reads were trimmed to a length of 100 bps for analysis using the Trim Fastq tool provided as part of rMATS package. Standard workflow for rMATS (default parameters as described in: http://rnaseq-mats.sourceforge.net/rmats3.2.1.beta/user_guide.htm) was used for the splicing analysis using the reads that cover the splicing junctions and target regions. Alternative splicing events with an FDR of < 0.05 were considered statistically significant.

### Polyadenylation site-enriched RNA-Seq

We performed polyadenylation site-enriched RNA-Seq with two methodologies (Gnomegen [89] and Nextera). Here we describe the second approach, Nextera. For polyadenylation site-enriched RNA-Seq, two different primary dermal fibroblasts, 12–1 and 12–3, were used as biological replicates. Proliferating, 7dCI, and siRNA-treated fibroblasts were lysed by adding 1 ml of Trizol per 10 cm plate and incubating the plate for 5 min at room temperature. RNA was isolated from the cell lysates using the Direct-zol™ RNA MiniPrep Plus kit (Zymo Research, Irvine CA) by following the manufacturer’s instructions. The concentration of RNA was measured using Nanodrop 2000c (Thermo Fisher Scientific). cDNA libraries containing fragments enriched for 3’UTR ends were created with the Nextera kit using the Smart-seq2 cDNA amplification method as described in [102]. Common forward primers were used for all samples; reverse primers with a unique barcode sequence (i5 indices) were specific for each sample. The size distribution of the cDNA library was confirmed using a High Sensitivity DNA chip (Agilent Technologies) on a Bioanalyzer 2100 Instrument (Agilent Technologies). Libraries with a uniform size distribution between 150 and 1000 bp were subjected to gel size selection to enrich for 180–280 bp sized fragments. The concentration of the final library was measured on a qubit fluorometer (Thermo Fisher Scientific). Single-end 150 bp reads were generated on an Illumina HiSeq 2500 Instrument. The sequencing reaction was run for 150 cycles.

### Polyadenylation site-enriched RNA-Seq analysis

Reads from polyadenylation site-enriched cDNA libraries were demultiplexed followed by removal of adapter and polyA tail sequences. Trimmed reads were aligned to the human genome (hg19/GRCh37 build) using TopHat (version 2.0.14) [94] using default parameters. Aligned reads were assigned to a polyadenylation site based on annotations in the Poly(A)site atlas (version:r1.0(hg19) by Gruber et al. [103] using the Perl script provided (http://www.polyasite.unibas.ch/). Only the polyadenylation sites annotated as TE (terminal exon), EX (any other exon except the terminal one), or IN (any intron), and with at least 10 counts across all the samples, were included for analysis. For genes containing two polyadenylation sites, the relative use of the distal polyadenylation site (RUD) [13, 18] was determined as distal polyadenylation counts/total read counts (distal plus proximal counts). The RUD values for two biological replicates were averaged to determine the RUD value of a gene. Changes in alternative polyadenylation between the two conditions were significant if the RUD difference between them was greater than 0.05. For genes with more than two polyadenylation sites, a parameter called relative site usage (counts for a polyadenylation site divided by total counts for all the polyadenylation sites) was calculated for all the polyadenylation sites of a gene. To perform differential expression analysis, counts from all the polyadenylation sites of a gene were combined and the combined counts for all the genes for two different conditions were subjected to DESeq2 (version 1.18) analysis [96, 104] using standard parameters (Ensembl annotation, GRCh37 assembly).

### Transcript decay rate measurements

Detailed protocols for cell culture and actinomycin D treatment are described in [63, 105]. Briefly, to monitor transcript decay rates, proliferating and 7dCI fibroblasts were treated with 15 μg/ml actinomycin D (Sigma-Aldrich, Inc., St. Louis, MO). Cells were washed with PBS and cell lysates were collected using Trizol reagent (Life Technologies) at 0, 120, 240, and 480 min after addition of actinomycin D. RNA was isolated from Trizol lysates using the Direct-zol™ RNA MiniPrep Plus kit (Zymo Research). cDNA library preparation, sequencing, and processing of reads were performed as described for polyadenylation-site enriched RNA-Seq.

### Decay rate calculations

For comparisons of decay rates under different conditions, only the genes with two polyadenylation sites (proximal and distal) in the 3′ UTR were used for analysis. Further, only transcripts with a minimum of 10 counts at *t* = 0 were used. For each polyadenylation site, the counts at four time points (0, 2, 4, and 8 h) were log-transformed and fit to a linear decay model ([63, 105]) using the least squares method to determine a fitting parameter (*R*^2^) and to obtain decay constants. Only the polyadenylation sites with R^2^ value greater than 0.6 were used. The decay constants (*k*) were converted to half-lives (ln2/*k*) for isoform-specific analysis.

### Motif analysis

For all of the transcripts that undergo APA with quiescence and had two detectable polyadenylation sites, sequences (in FASTA format) were obtained from the UCSC Genome Browser (Table browser tool, hg19/GRCh37 build, accessed on March 2018) that include the polyadenylation site itself, 100 nts upstream (for UGUA motif analysis), and the region 20 to 40 nt downstream (for U-rich and UG-rich motif analysis) of the polyadenylation site. For hexamer analysis, the hexamer associated with each of the polyadenylation sites was obtained from Poly(A)site atlas annotations (*Homo sapiens*-version:r1.0(hg19)) by Gruber et al. (http://www.polyasite.unibas.ch/) [103]. For sites associated with more than one hexamer, we chose the hexamer with the highest signal strength as determined by Gruber et al. For UGUA analysis, FIMO (v4.12.0) [106] motif analysis tool of the MEME suite was used with *p* value set to 1 to return matches to all of the UGUA motifs. Post-processing of the FIMO results was used to check for exact matches. For RBP motif analysis, primary sequences (in FASTA format) from the alternate region (region between proximal and distal sites in the 3′ UTR) for genes that become longer (distal polyadenylation site use) with quiescence were extracted using the Table browser tool of the UCSC Genome Browser (hg19/GRCh37 build, accessed on March 2018). To generate a background dataset, all the sequences from alternate regions of genes that use more proximal sites with quiescence and genes with no change in polyadenylation site use with quiescence were used. RBP motifs enriched in primary sequences in comparison with background sequences were obtained using the analysis of motif enrichment (AME, v4.12.0) motif enrichment tool [107] of the MEME suite. The RNA motifs from Ray2013 *Homo sapiens* motif database [108] were used for enrichment testing. Only the RBP motifs enriched in both 12–1 and 12–3 biological replicates were considered. For U-rich and UG-rich analysis, the sequences of the regions encompassing 20 to 40 nt downstream of the polyadenylation site for each gene were extracted for all genes with two polyadenylation sites using the Table browser tool of the UCSC genome browser (hg19/GRCh37 build, accessed on March 2018). The U-rich sequences in this region have been shown to be the preferred binding sites of CstF64 using crosslinking immunoprecipitation (CLIP)-Seq analysis [109]. Percent U was calculated by determining the fraction of Us present in this region. Percent UG was calculated by determining the sum of the fractions of Us and Gs present in this region. For analysis of 4-mer UUUU sequence [110], the presence or absence of a UUUU motif was determined in this region.

### Splicing site analysis

Nucleotide sequences were extracted for the 5′ and 3′ splice sites for 139,180 constitutive exons from HEXEvent online database [111] and for the introns called differentially retained (FDR < 0.05) by rMATS in proliferating or quiescent fibroblasts (Additional file 4). For analyzing 5′ and 3′ splice sites, motifs of 9 bases (3 bases in the exon and 6 bases in the intron) and 23 bases (20 bases in the intron and 3 bases in the exon), respectively, were used. A position weight matrix was generated from constitutive exon 5′ and 3′ sequences using scripts written in the R programming language [112, 113]. Based on this position weight matrix, the probability of each sequence was determined for each sequence in the list of constitutive exons, introns retained in proliferating conditions and introns retained in quiescent conditions. Statistical significances of the groups of probabilities were determined with ANOVA with Tukey’s multiple comparison test. Sequence logos were generated from the position weight matrix using the R programming language (seqLogo package, https://bioconductor.org/packages/release/bioc/html/seqLogo.html) [114].

### Antibodies for immunoblotting

Antibodies against tubulin (T6074) and CFIm25 (AV40695-100UG, 1:800 dilution) were obtained from Sigma-Aldrich, Inc. (Saint Louis, MO). An antibody against CstF-64 (sc-28201, 1:200) was purchased from Santa Cruz Biotechnology, Inc. (Dallas, TX). An antibody against U1-70K (06-1297, 1:2000) was purchased from EMD Millipore (Billerica, MA). Antibodies against CPSF73 (A301-090A-T), U2AF65 (A303-665A-T), FUS (A300-292A-T), and RNA Polymerase II Phospho S5 (A304-208A-T) were purchased from Bethyl Laboratories (Montgomery, TX) and used at 1:1000 dilution.

### Immunoblotting

Immunoblotting was performed using a standard protocol similar to that described previously [7]. Briefly, cells were lysed using mammalian protein extraction reagent (MPER) (Thermo Fisher Scientific Inc., Waltham, MA) containing protease and phosphatase inhibitors (Roche Applied Science, Indianapolis, IN) according to the manufacturer’s instructions (Thermo Fisher Scientific Inc.). Total protein concentrations in collected lysates were measured using Pierce™ BCA protein assay kit (Thermo Fisher Scientific Inc.). Samples were run on SDS PAGE gels and transferred to polyvinylidene difluoride Immobilon-P membranes (EMD Millipore, Billerica, MA). Membranes were blocked with 5% BSA in phosphate-buffered saline-Tween. Immunodetection was performed using primary and HRP-conjugated secondary antibodies based on standard protocols.

### Mouse wounding assays

All experiments were approved by the UCLA Office for Animal Research, protocol number 2015–033. C57/BL6 mice were provided housing and husbandry in accordance with Institutional Animal Care and Use Committee approved protocols. Mice that were approximately 8–10 weeks of age were anesthetized, shaved, and provided with analgesia. We introduced one full thickness dermal punch biopsy of 3.5 mm on each mouse’s upper back. On day 5 after wounding, the mouse was 83.6% healed. Mice were euthanized with CO_2_ followed by cervical dislocation. We excised the wound bed *en bloc* with the surrounding soft tissue and at least 0.5 cm of normal tissue surrounding the incision. We also collected normal skin from the same mice for comparison. Skin and wounds were fixed in formalin and paraffin-embedded. Slides were cut from paraffin blocks for immunohistochemistry.

### Immunohistochemistry

Tissue slices (4 μm) from paraffin-embedded blocks containing wounds were de-paraffinized and rehydrated with a graded series of alcohols. Slides were subjected to heat-induced antigen retrieval with pH 6.0 citrate buffer. Slides were treated with primary antibodies against Ki-67 (Abcam, catalog no. ab16667, dilution 1:150), histone H4 (EMD Millipore, 05-858, 1:2000), CstF-64 (Bethyl Laboratories, IHC-00221, 1:1000), CPSF73 (Bethyl, A301-090A, 1:200) or CFIm25 (Sigma, AV40695, 1:200), followed by EnVision+ HRP-conjugated secondary antibody (Dako) and DAB chromogen (Roche) visualization. Slides were counterstained with hematoxylin and imaged with a Zeiss AXIO Imager.D2 microscope.

### Immunofluorescence

A monolayer of contact-inhibited fibroblasts in a 35-mm dish with a glass bottom (MatTek Corporation, Ashland, MA) was scratched (crosswise) using a sterile 1 ml pipette tip to create a region free of cells (wound area). The cells were then gently washed two times using complete medium to remove the non-adherent cells generated during scratching. After 24 h, the cells were fixed with 4% paraformaldehyde (Santa Cruz Biotechnology Inc., Dallas, TX) in PBS for 15 min at room temperature and then washed three times with ice-cold PBS. The cell permeabilization was performed using 0.25% Triton X-100 (Thermo Fisher Scientific, NJ) followed by washing the cells three times with PBS. The cells were blocked using blocking solution (1% bovine serum album (BSA) in PBS containing 0.2% Tween (Thermo Fisher Scientific) at room temperature for 30 min. After blocking, the cells were incubated with primary antibodies (CstF64, CPSF73, or CFIm25) in blocking solution (1:100 dilution) at 4 °C in a humidified chamber overnight. The cells were then washed three times with PBS followed by incubation with Alexa-488 labeled secondary antibody (Thermo Fisher Scientific) at 1:250 dilution for 1 h at room temperature. After washing the cells three times with PBS, the cells were stained with DAPI using the VECTASHIELD hardset antifade mounting medium with DAPI (Vector Laboratories, Inc., Burlingame, CA). The images were taken at 10X magnification on a Zeiss confocal microscope (LSM 710, Carl Zeiss). Images were analyzed using *ImageJ* (v1.52a).

### siRNA transfection

siRNAs against CFIm25 and CPSF73 were purchased from Sigma-Aldrich. siRNAs against CstF-64 were purchased from Sigma-Aldrich (CstF64.1) and Origene Technologies Inc., Rockville, MD (CstF64.2 and CstF64.3). siRNAs were transfected into fibroblasts or cancer cells using GeneMute transfection reagent from SignaGen Laboratories (Rockville, MD) according to the manufacturer’s instructions.

### Real-time PCR

For real-time PCR, DNA primers were designed with Primer3 for UBC primers or NCBI Primer-BLAST for all other primers, and synthesized by Integrated DNA Technologies (Coralville, IA). RNA was isolated using the PureLink RNA Kit (Thermo Fisher Scientific). cDNA was treated with TURBO DNA-*free*™ Kit (Thermo Fisher Scientific) to eliminate the remaining DNA. Real-time PCR was performed with SYBR® Green One-Step Real-Time RT PCR Kit (Thermo Fisher Scientific). Samples were cycled on a BioRad CFX96 Real Time PCR instrument driving a Biorad C1000 Thermal Cycler for 40 cycles. The ΔΔCt method was used to determine the abundance of different PCR products [115]. Values for each gene of interest were normalized to UBC for the same sample. Primer sequences were as follows: CstF64, 5’-GCAAGCTTCTATGCAGGGTG-3′ and 5′-TTGCATCGGCACTTGAACTC-3′; CPSF73, 5′-GAAGTCGAGGGGAGGAGTCT-3′ and 5′-AGCTCCAAGGGGTCGGAT-3′; CFIm25, 5′-GCACCATCAACCTGTACCCTC-3′ and 5′-AGTAACACATGGGGTAGCCG-3′; long INF2, 5′-GGAGGAGGTGTGTGTCATCG-3′ and 5′-CTCCTGCAGGGTTACTGGTG-3′; short INF2, 5′-GCTGCGGAACGAGTTTATCG-3′ and 5′-GGAGGTGCTGCTTAGGTGAG-3′; long BOC, 5′-TCAGCAACGTGATGATCTGTGA-3′ and 5′-CCGCTCTATGGTTTCAGGAAGG-3′; short BOC 5′-CCTCATCTCTCCCACCCTGAA- 3′ and 5′-TGAGGTTTTCCAAGGGCACAA-3′, UBC, 5′-TCTTGTTTGTGGATCGCTGTGA-3′ and 5′-CAGGAGGGATGCCTTCCTTATC-3′.

### Incucyte in vitro wound healing assays

For wound healing assays, fibroblasts were plated in the wells of an Incucyte™ ImageLock™ 96-well plate (Essen BioScience) and the WoundMaker™ tool was used to create a denuded area in each well on the plate. The IncuCyte™ ZOOM live-cell analysis system (Essen BioScience) was used to automatically collect time-lapse images (phase-contrast) and to quantify cell migration over time as the density of cells in the denuded area relative to the density of cells out of the denuded area (relative wound density). Plots were determined to be statistically significantly different based on repeated measures two-way ANOVA with Dunnett’s multiple comparison test.

### Statistical analyses and plots

Statistical significance determinations were performed with two-tailed tests for all analyses. For DESeq/DESeq2, splicing, and DEXSeq, the software included multiple hypothesis testing correction. All errors bars represent standard deviations. For the Wilcoxon test, we checked whether the data were normally distributed. We used Fisher’s exact tests when sample sizes were low. Statistical significance for t-tests was determined using Prism (6.0f, GraphPad Software, La Jolla, CA). Statistical significance for correlations were performed using the cor() function in R. The hypergeometric test was performed with dhyper() function in R. The Wilcoxon test was performed with the Wilcox.test() function in R. Time series analysis for migration assays was performed with Prism. All bar graphs for RT-PCR and plots for migration assays were performed in Prism. All box plots and density plots were generated with ggplot2 package [116]. Plots for motif frequencies were generated in Prism.

## Additional files

Additional file 1: Supplementary figures and supplementary **Tables S1-S5**. (PDF 6052 kb)

Additional file 2: Expression of polyadenylation factors with quiescence. (XLSX 14 kb)

Additional file 3: Alternative isoform use with quiescence. (XLS 1401 kb)

Additional file 4: Gene Ontology for alternative isoform use. (XLSX 932 kb)

Additional file 5: Alternative splicing with quiescence. (XLSX 136 kb)

Additional file 6: Gene Ontology for alternative splicing. (XLSX 520 kb)

Additional file 7: Polaydenylation site use with quiescence. (XLS 194 kb)

Additional file 8: Polyadenylation site use with quiescence for genes with more than two polyadenylation sites. (XLSX 537 kb)

Additional file 9: Gene Ontology for alternative polyadenylation. (XLSX 618 kb)

Additional file 10: Alternative polaydenylation in knockdown cells. (XLS 309 kb)

Additional file 11: Differential expression with quiescence and knockdown. (XLSX 160 kb)

Additional file 12: Isoform-specific half-lives with quiescence. (XLSX 40 kb)

## Abbreviations

7dCI: 7 days of contact inhibition
7dSS: 7-day serum-starved
APA: Alternative polyadenylation
BAM: Binary version of a SAM file
BCA: Bicinchoninic acid assay
BOC: Brother of CDO
CFIm25: Nudix (nucleoside diphosphate linked moiety X)-type motif 21
CLIP: Crosslinking immunoprecipitation
CLUAP1: Clusterin associated protein 1
CPSF: Cleavage and polyadenylation specificity factor
CSTF: Cleavage stimulation factor
CTD: Carboxy terminal domain
DAB: 3,3′-Diaminobenzidine
DMEM: Dulbecco’s modified Eagle medium
FAK: Focal adhesion kinase
FBS: Fetal bovine serum
FDR: False discovery rate
FUS: Fused in sarcoma
GO: Gene ontology
GSEA: Gene set enrichment analysis
GSEMA: Gene Set Enrichment Made Awesome
HER2: Human epidermal growth factor receptor 2
HRP: Horse radish peroxidase
IGV: Integrated Genome Viewer
INF2 Inverted Formin: FH2 and WH2 domain containing
MEME: Multiple Em for Motif Elicitation
MPER: Mammalian protein extraction reagent
NMD: Nonsense-mediated decay
P: Proliferating
PAGE: Polyacrylamide gel electrophoresis
PPIH: Peptidylprolyl isomerase H
PRPF4: Pre-MRNA Processing Factor 4
rMATS: Replicate Multivariate Analysis of Transcript Splicing
RUD: Relative use of the distal polyadenylation site
SDS: Sodium dodecyl sulfate
TRA2β: Transformer-2 protein homolog beta
U1-70K: U1 small nuclear ribonucleoprotein 70K
U2AF2/U2AF65: U2 Small Nuclear RNA Auxiliary Factor 2
UBC: Ubiquitin C
UR APA: Upstream region APA or alternative polyadenylation affecting at least one polyadenylation site in the coding sequence
UTR APA: Alternative polyadenylation affecting polyadenylation sites in the UTR
WASp: Wiscott-Aldrich Syndrome protein
