# Supplementary material for: Alternative polyadenylation factors link cell cycle to migration
Source: Genome Biol. 2018 Oct 25;19:176. doi: 10.1186/s13059-018-1551-9 (PMC6203201; doi:10.1186/s13059-018-1551-9)
Supplement: Supplementary file 1 — Supplementary figures and supplementary Tables S1-S5. (PDF 6052 kb) [file 13059_2018_1551_MOESM1_ESM.pdf]

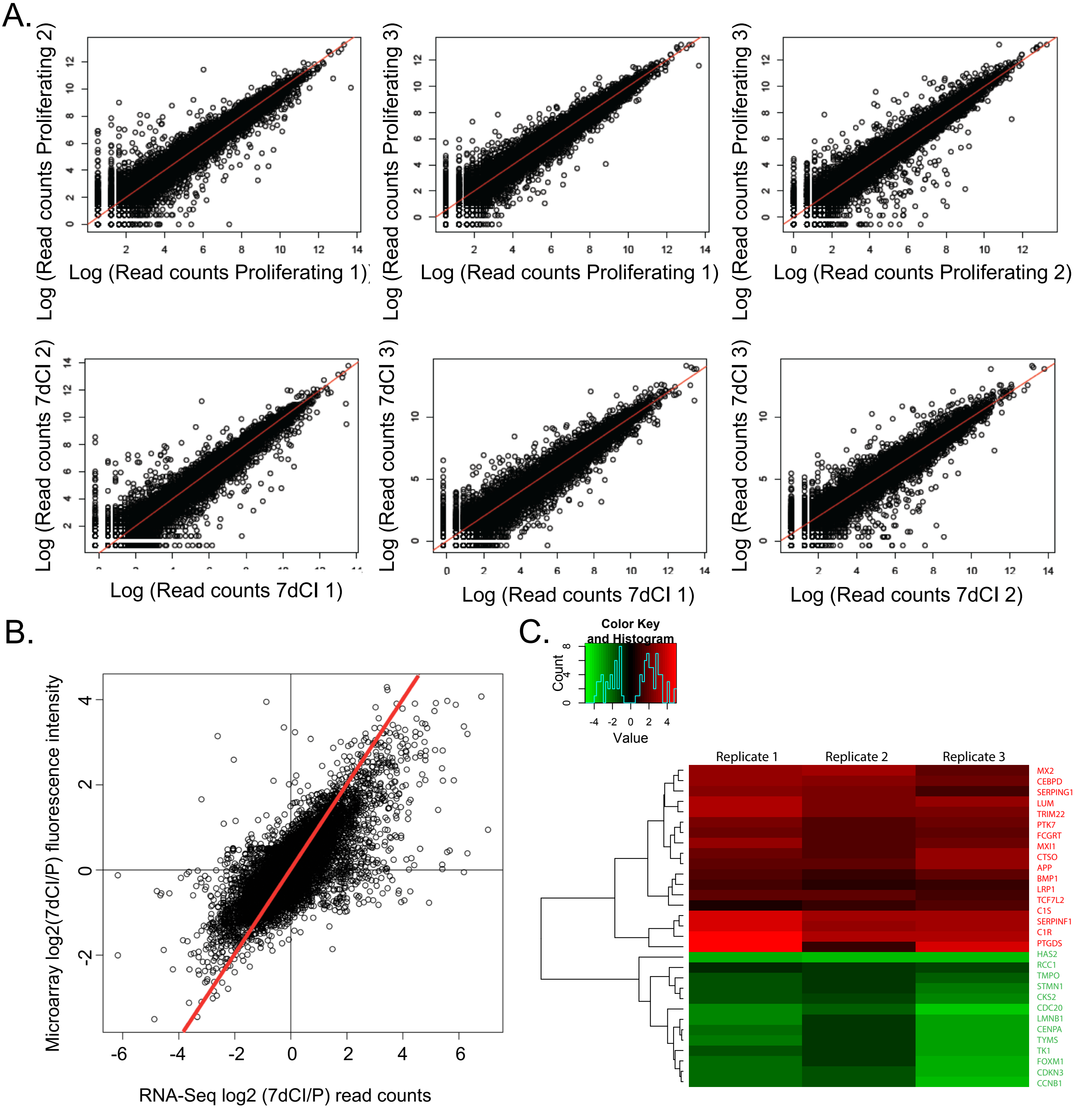


**Fig S1. RNA-Seq data are reproducible, similar to microarray analysis of the same samples, and consistent with previous studies.** (A) RNA-Seq read counts in biological replicates of proliferating and contact-inhibited (7dCI) fibroblasts. The log of the read count in one biological replicate is mapped on the x-axis and a different biological replicate is mapped on the y-axis. R squared values comparing biological replicates are 0.94 or higher for proliferating samples and 0.83 or higher for 7dCI. (B) A scatterplot depicting fold-change in expression by RNA-Seq ($log_2$(7dCI/P normalized read counts)) on the x-axis and microarray on the y-axis ($log_2$(7dCI/P fluorescence intensity values)) is shown (r = 0.75, *p*-value < 0.001). The red line indicates the line y=x. (C) Genes previously identified as consistently regulated in multiple different quiescence conditions [9] that also change in RNA-Seq data reported here are shown in three independent comparisons of proliferating versus 7dCI fibroblasts. Heatmap intensities represent $log_2$ ratio of normalized 7dCI/proliferating RNA-Seq counts. Red indicates upregulation with quiescence; green indicates downregulation. Gene name colors correspond to expression changes in Coller et al. (2006). The dendrogram resulting from hierarchical clustering of the expression data is provided.

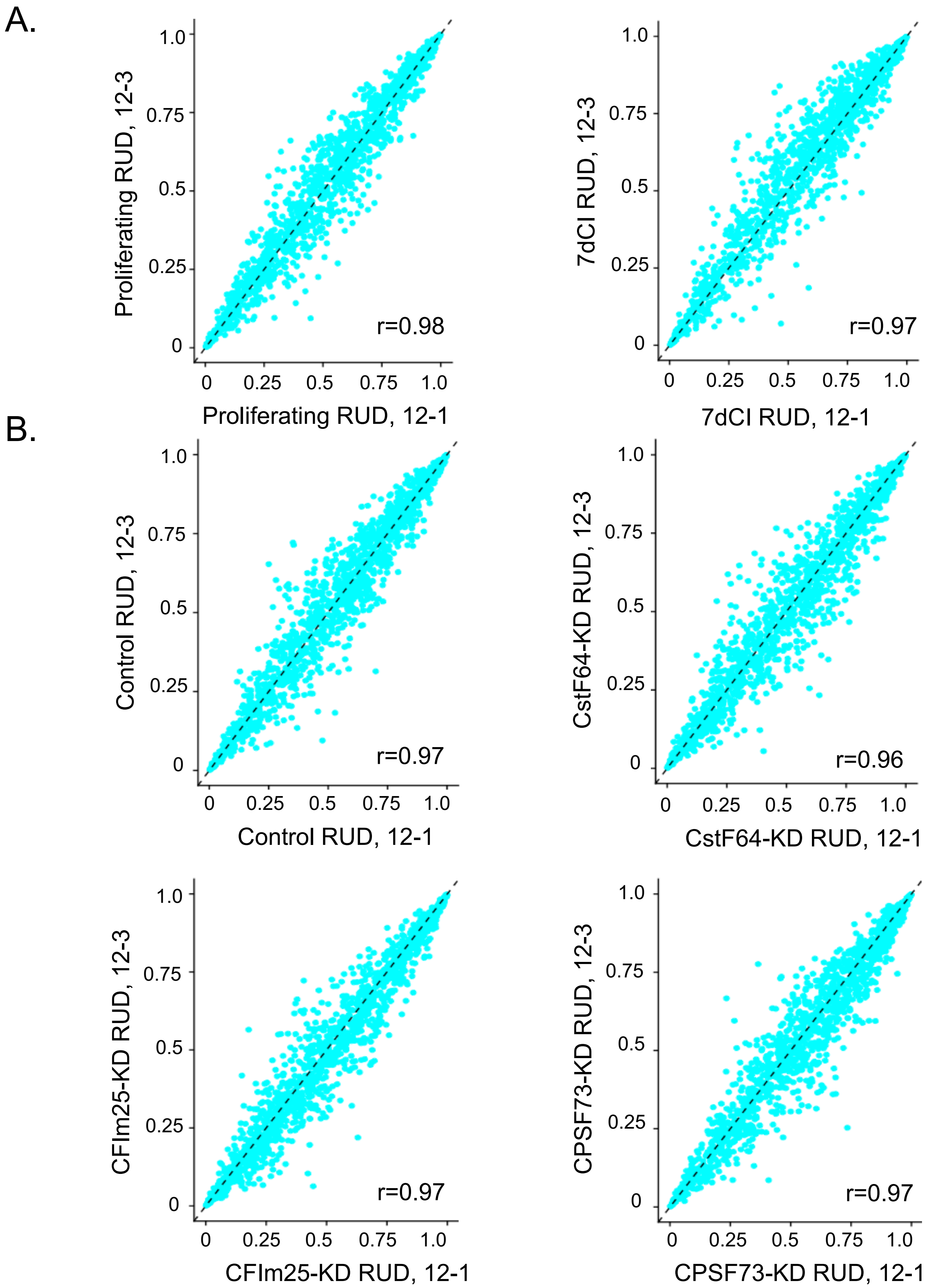


**Fig S2. Polyadenylation site-enriched RNA-Seq data are reproducible.** (A) The relative use of the distal polyadenylation site (RUD) was determined for proliferating conditions and 7dCI conditions for two independent experiments. The RUD for one example of proliferating cells is plotted on the x-axis and the RUD for another proliferating sample is plotted on the y-axis (left panel). Similar plots are shown for 7dCI states (right). Correlation values (r, Pearson correlation) are reported for each plot. (B) Relative use of the distal polyadenylation site for each siRNA (control, CPSF73, CstF-64, CFIm25) for each gene in 12-1 (x axis) versus 12-3 (y axis) fibroblasts. Correlation values (r, Pearson correlation) are reported for each plot.

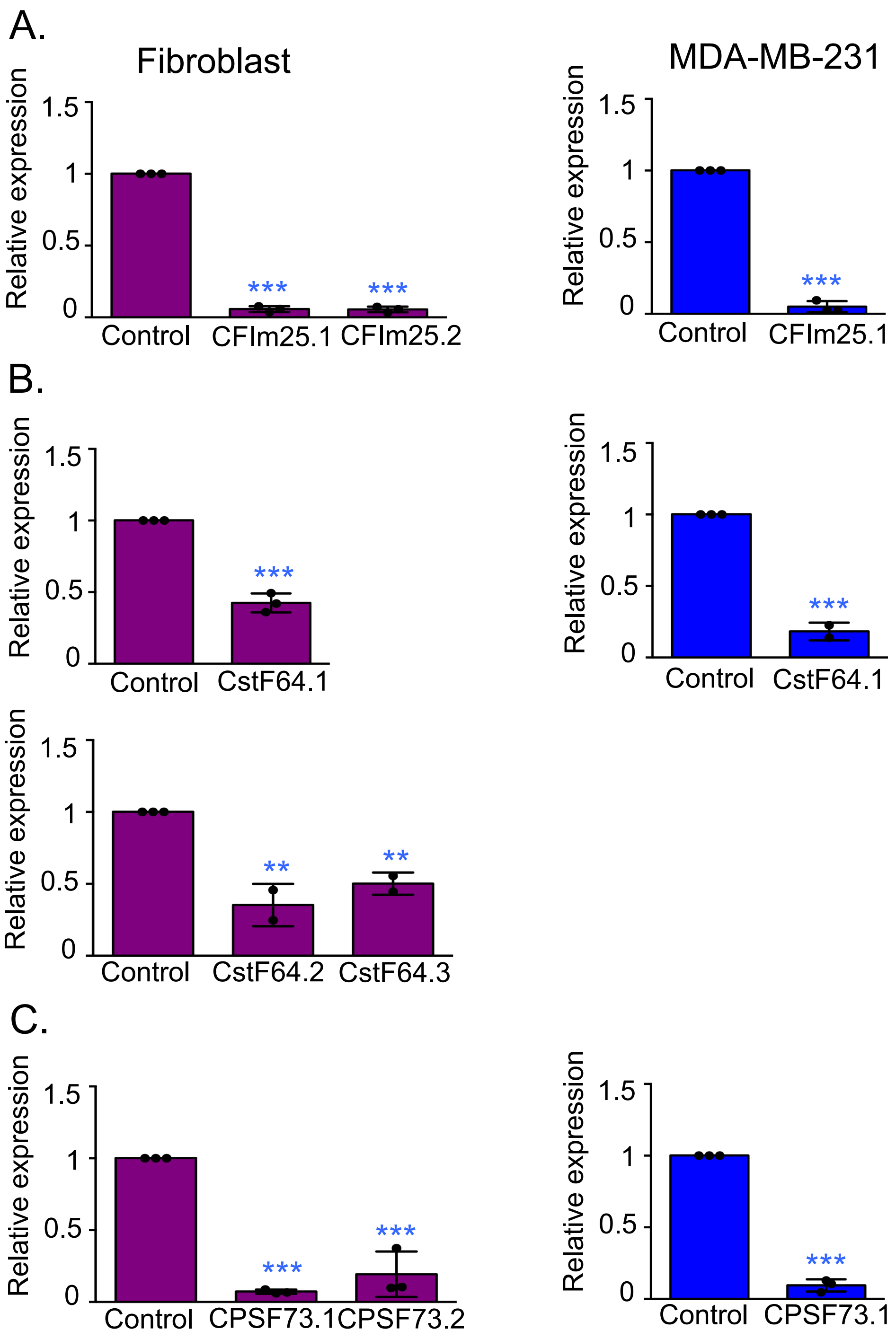


**Fig S3. siRNAs against CFIm25, CstF-64, or CPSF73 result in reduced expression of the targeted APA factor.** Fibroblasts or triple negative breast cancer (MDA-MB-231) cells were transfected with a control siRNA or an siRNA against (A) CFIm25, (B) CstF-64, or (C) CPSF73. Levels of the targeted APA factor were monitored in the transfected cells with real-time PCR. Plots show individual datapoints as dots. Bar graphs represent mean and average +/- S.D. with control siRNA normalized to 1. For fibroblasts 12-1, the number of replicates for CFIm25 and CPSF73 is 3. The number of replicates for CstF-64-1 is 3. The number of replicates for CstF-64-2 and CstF-64-3 is 2. For MDA-MB-221, the number of replicates for CFIm25 and CPSF73 is 3. The number of replicates for CstF-64 is 2. Statistical significance in knockdown cells compared to control cells was determined with two-tailed, unpaired t-tests.

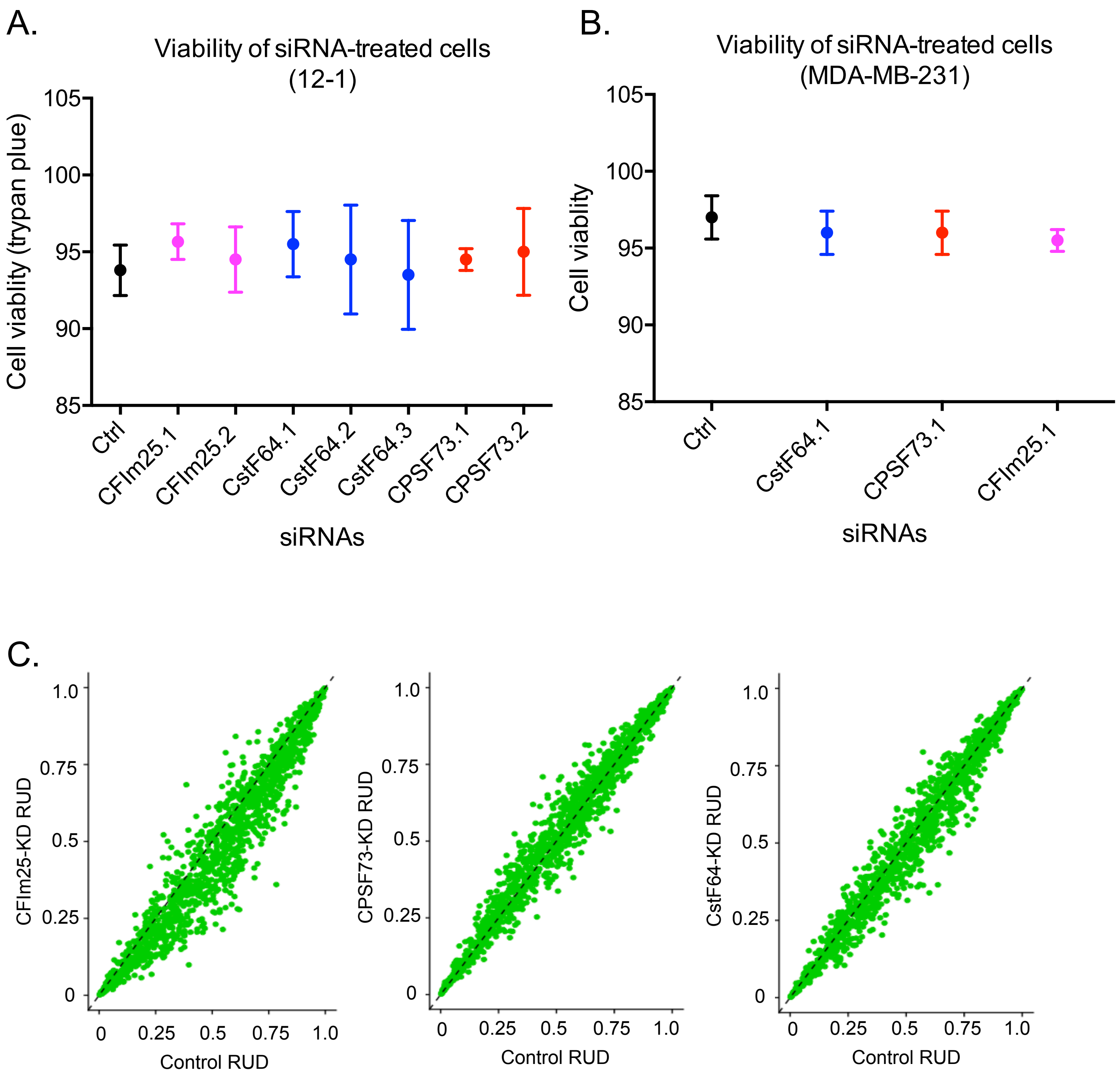


**Fig S4. siRNAs against CFIm25, CstF64 or CPSF73 do not affect cell viability.** (A) Primary human dermal fibroblasts were transfected with a control siRNA or an siRNA against CFIm25, CstF64 or CPSF73. Cell viability was monitored with trypan blue. (B) Viability was monitored with trypan blue in triple negative breast cancer cells transfected with a control siRNA or an siRNA against CstF64, CPSF73 or CFIm25. (C) Effect of knockdown of polyadenylation factors on polyadenylation site selection. Fibroblasts transfected with siRNAs against CFIm25, CPSF73 or CstF-64, or a control siRNA, were analyzed with polyadenylation site-enriched RNA-Seq. The relative use of the distal isoform (RUD) for each gene is plotted on the x-axis and the RUD for the same gene in knockdown cells is plotted on the y-axis.

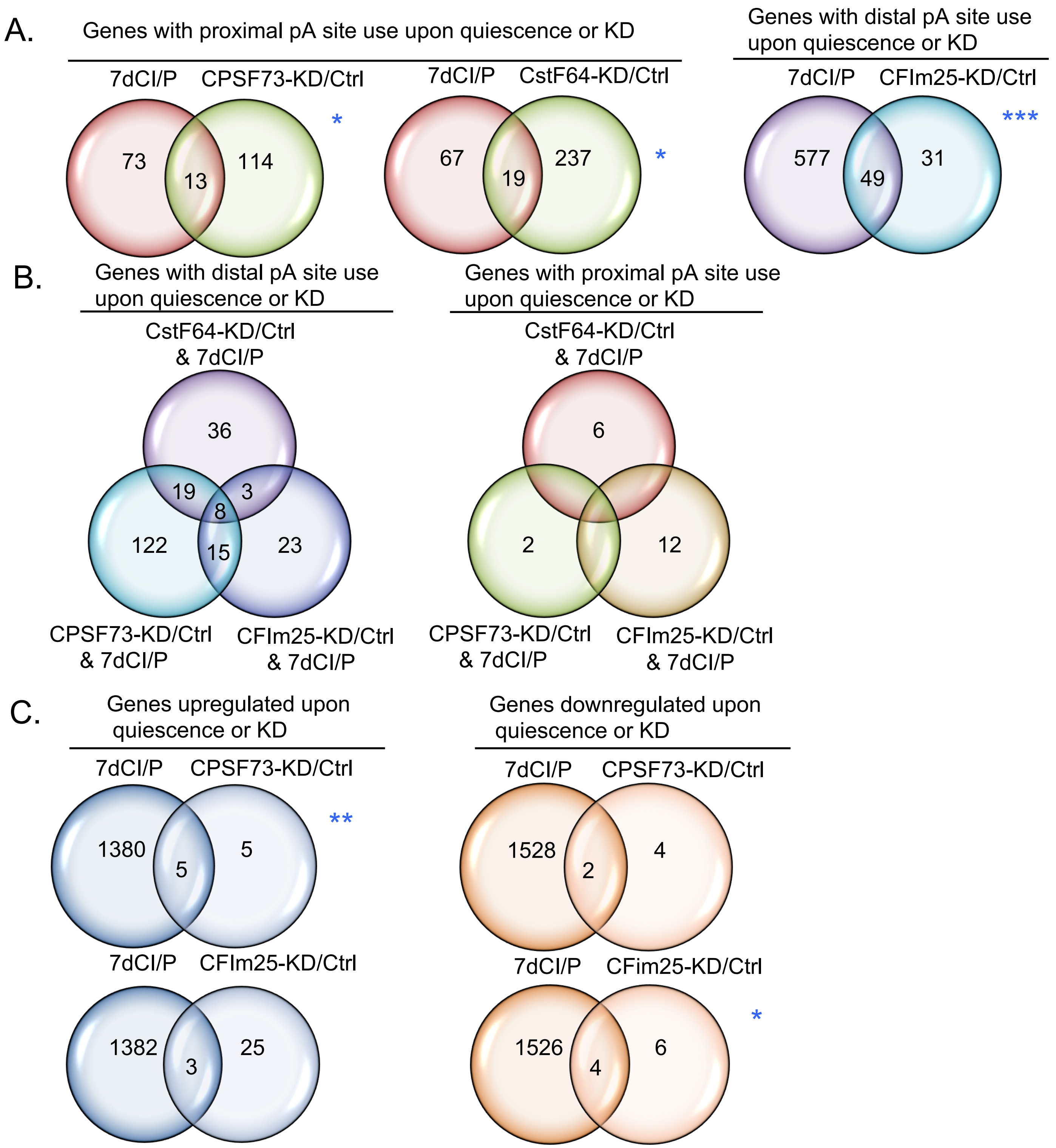


**Fig S5. Overlap among changes in polyadenylation site selection and gene expression with quiescence and cleavage and polyadenylation factor knockdown.** (A) Overlap between genes that change polyadenylation site selection with quiescence and genes that change polyadenylation site selection with cleavage and polyadenylation factor knockdown. Overlap is shown for genes that shift to more proximal polyadenylation site use with quiescence and knockdown of CPSF73 or CstF64, and overlap between genes that shift to more distal polyadenylation site use with quiescence and knockdown of CFIm25. (B). Overlap is shown among genes that shift to greater use of a distal polyadenylation site with quiescence and knockdown of a polyadenylation factor. Overlap is also shown among genes that shift to greater use of a proximal polyadenylation site with quiescence and knockdown of a polyadenylation factor. (C) Overlap among genes that change expression in quiescence and that change expression with knockdown of CPSF73 or CFIm25.

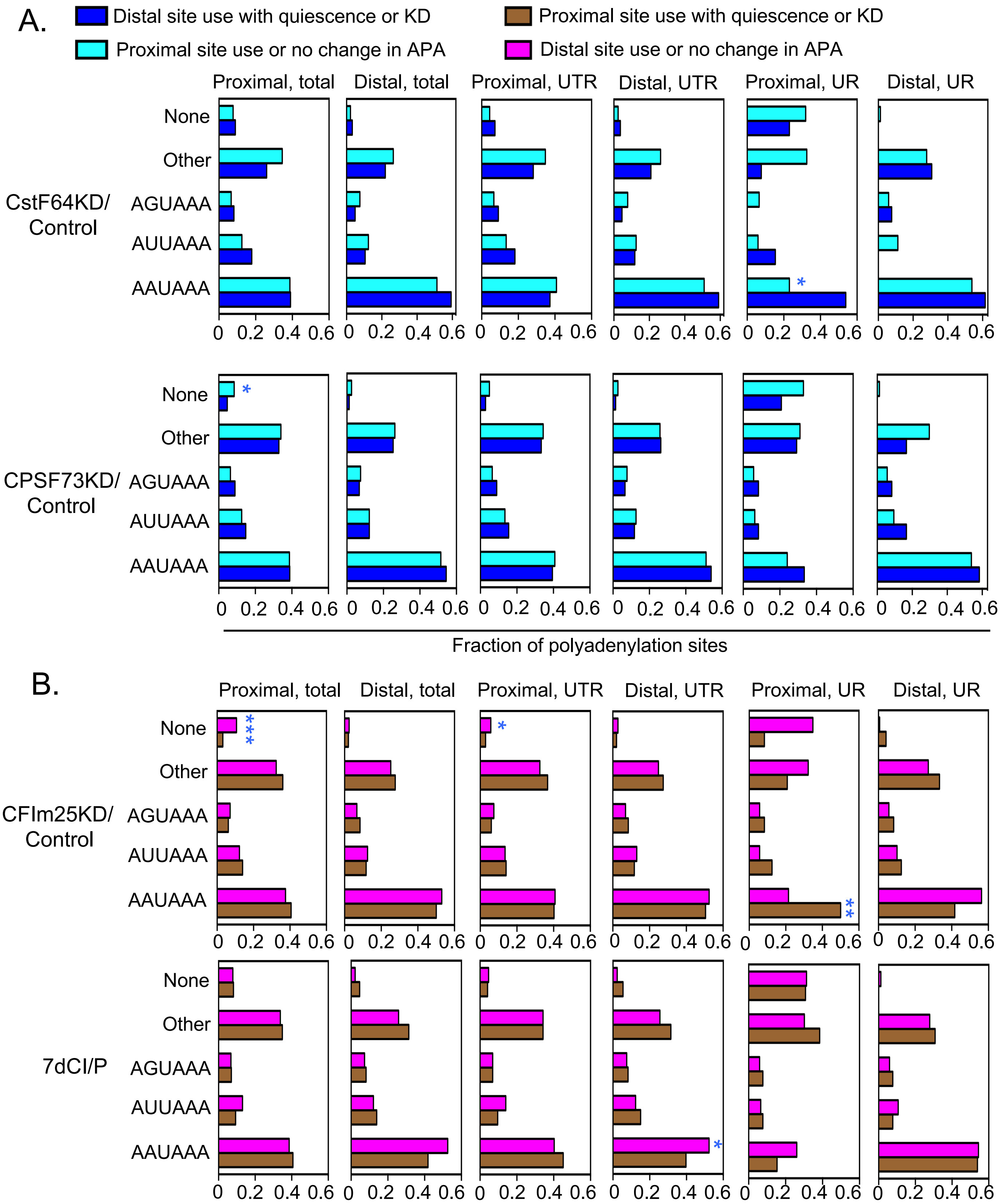


**Fig S6. Frequency of different hexamers in genes that shift polyadenylation site use with quiescence or cleavage and polyadenylation factor knockdown.** (A) Frequency of different hexamers for genes that shift to greater use of distal polyadenylation sites with CstF64 or CPSF73 knockdown (dark blue) compared with genes that do not shift to greater use of distal polyadenylation sites (light blue (B) Hexamer frequency data are provided as in (A) for cells with CFIm25 knockdown or transitioning to quiescence for genes that shift to greater use of proximal polyadenylation sites (brown) or a control set of genes that do not shift to greater use of proximal sites (pink).

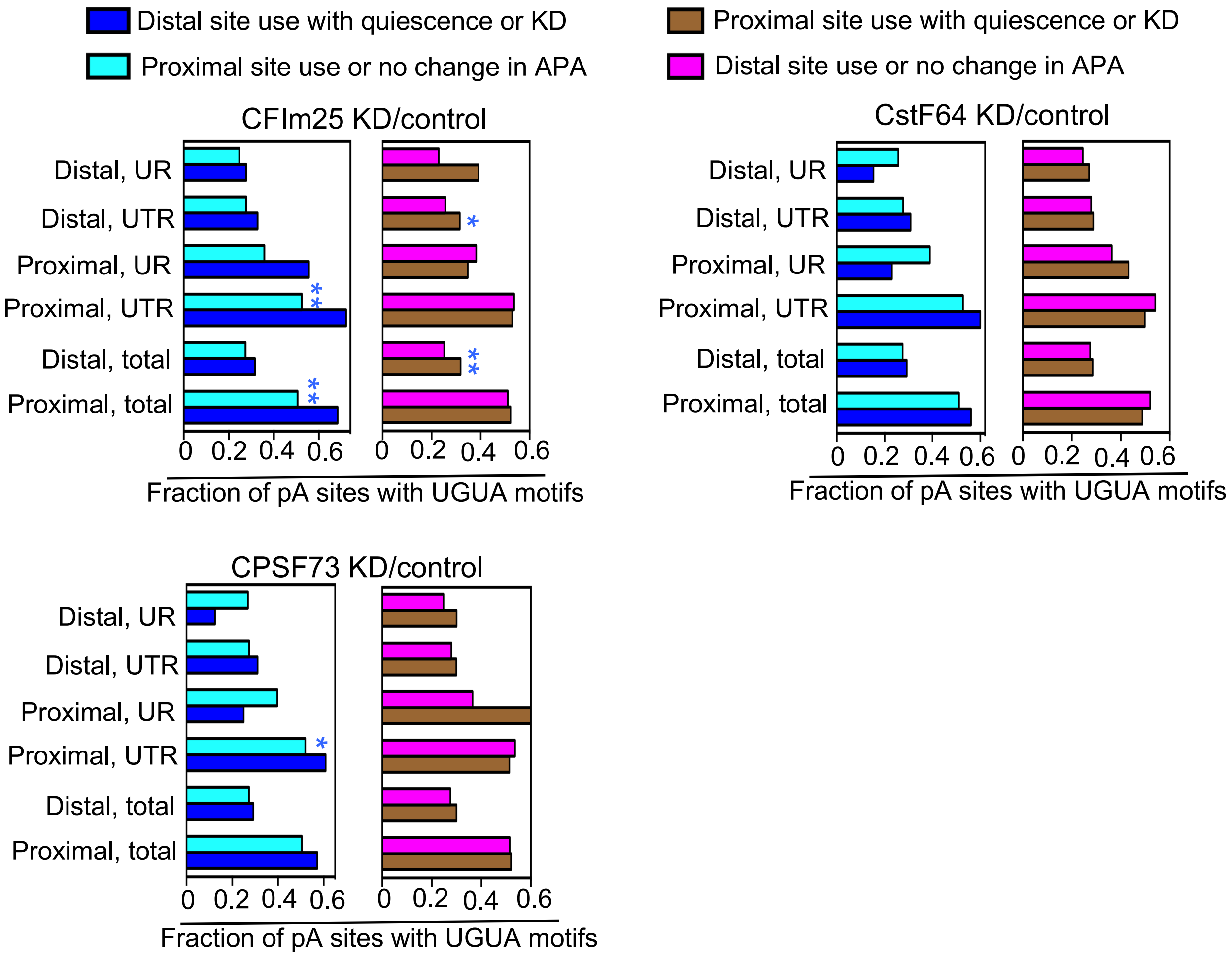


**Fig S7. Frequency of UGUA upstream of hexamers in genes that shift polyadenylation site selection with cleavage and polyadenylation factor knockdown.** (A) The fraction of polyadenylation sites with UGUA motifs is plotted for genes that shift to greater use of distal polyadenylation sites (dark blue) and control genes (light blue) and genes that shift to greater use of proximal polyadenylation sites (brown) and control genes (pink). Data are shown for fibroblasts transfected with siRNAs against CFIm25, CstF64 or CPSF73 compared with a control siRNA. Data are provided for all distal polyadenylation sites, all proximal polyadenylation sites, distal polyadenylation sites for UTR APA, proximal polyadenylation sites for UTR APA, distal polyadenylation sites for UR APA, and proximal polyadenylation sites for UR APA. Statistical significance was determined by two-tailed Fisher's exact test.

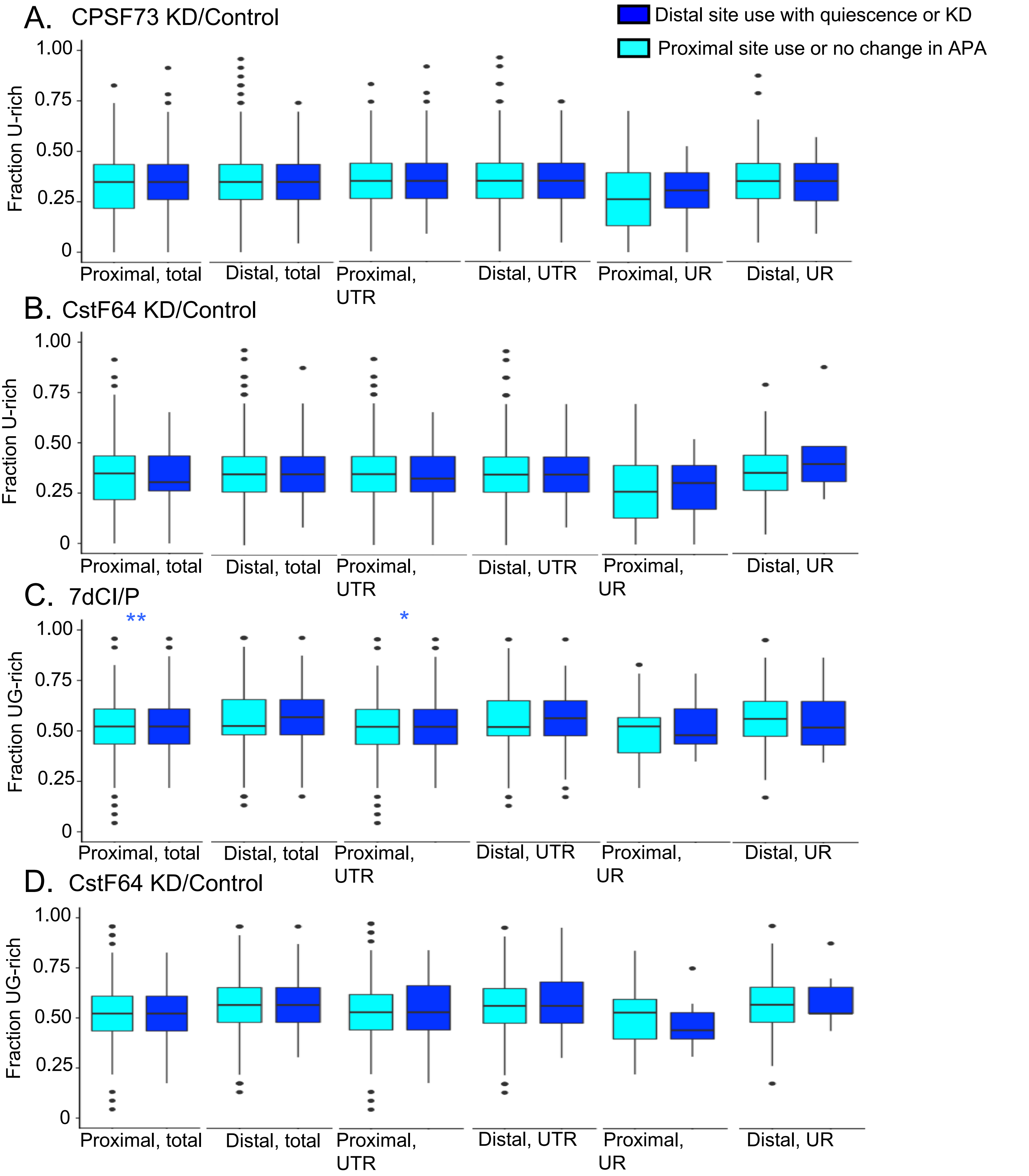


**Fig S8. Fraction of bases between 20 and 40 bps downstream of the polyadenylation site that are U or G.** The fractions of nucleotides that are uracils (U-rich) between 20 and 40 bps downstream of the hexamer are plotted for proximal and distal polyadenylation sites. Data are provided for genes that shift to greater use of distal polyadenylation sites (dark blue) and control genes (light blue) for fibroblasts transfected with siRNAs against CPSF73 (A) or CstF64 (B) compared with a control siRNA. The same analysis was also performed for the combined fraction of uracils and guanines (UG-rich) comparing contact-inhibited with proliferating fibroblasts (C) and comparing fibroblasts transfected with a siRNA against CstF64 with fibroblasts transfected with a control siRNA (D). Statistical significance by Wilcoxon signed-rank test.

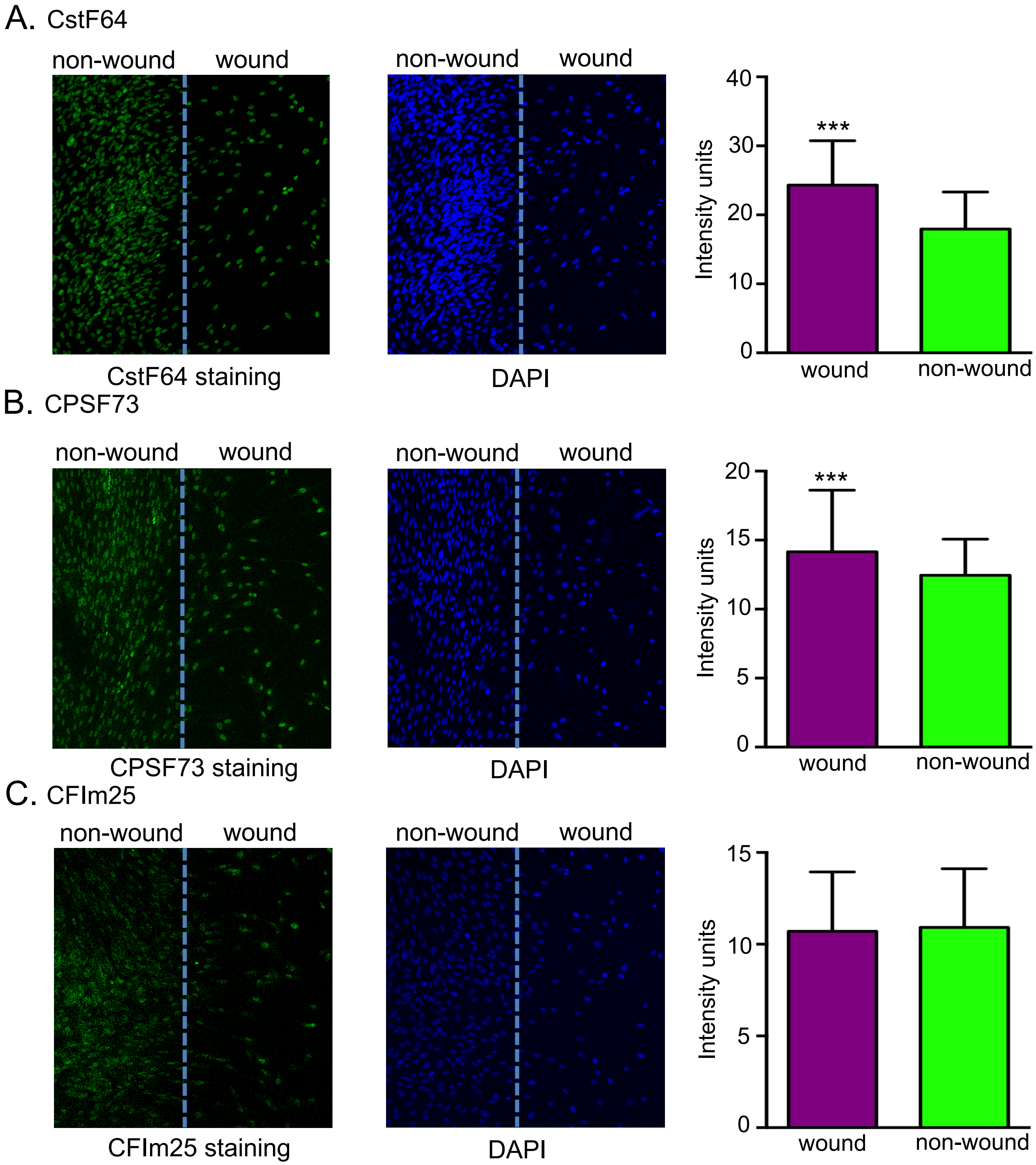


**Fig S9. Levels of polyadenylation factors in migratory vs. non-migratory fibroblasts.** Fibroblasts were seeded on a 35 mm dish with a glass bottom and allowed to grow until contact-inhibited. A pipet tip was used to denude a portion of the plate. Cells were analyzed with immunofluorescence (green, left panels) for the levels of cleavage and polyadenylation factors CstF-64 (A), CPSF73 (B), and CFIm25 (C). Cells were also stained with the DAPI nuclear stain (middle panels). Levels of each factor in the migratory cells (in wound area) and non-migratory cells (non-wound area) were quantified with *ImageJ* (right panels). Bar graphs represent mean and +/- S.D. The green intensity in the nuclear region was considered for analysis. (A) For CstF64 staining, 95 cells in the wound area and 295 cells in the non-wound area were used for analysis. (B) For CPS73 staining, 99 cells in the wound area and 354 cells in the wound area were used for analysis. (C) For CFIm25 staining, 74 cells in the wound area and 320 cells in the non-wound area were considered. Statistical significance was determined with two-tailed, unpaired t-tests. The figure shows representative results from 2 (CPSF73 and CFIm25) or 3 (CstF-64) independent experiments.

**Table S1. Number of RNA-Seq reads for each biological replicate and proliferative state.**

| | Proliferating | | | 7-day Contact Inhibited | | |
|---|---|---|---|---|---|---|
| Biological Replicate | 10-5 | 12-1 | 12-1 | 10-5 | 12-1 | 12-1 |
| Sequencing Run | 1 | 2 | 3 | 1 | 2 | 3 |
| Reads Matched to barcodes | $3.4 \times 10^7$ | $6.8 \times 10^7$ | $3.5 \times 10^7$ | $3.6 \times 10^7$ | $6.1 \times 10^7$ | $5.0 \times 10^7$ |
| Total Reads in Run | $1.1 \times 10^8$ | $1.1 \times 10^8$ | $1.4 \times 10^8$ | $1.4 \times 10^8$ | $1.4 \times 10^8$ | $3.2 \times 10^8$ |

**Table S2. Top genes undergoing differential expression with quiescence**

| Official Gene symbol | Gene description | $Log_2$(quiescent counts/ proliferating counts) | DESeq qvalue | Gene Ontology |
|---|---|---|---|---|
| Genes most strongly downregulated with quiescence | | | | |
| KISS1 | KiSS-1 metastasis-suppressor | -6.4 | 3.6E-11 | Cytoskeletal organization; protein binding |
| ACTC1 | actin, alpha, cardiac muscle 1 | -6.2 | 2.7E-02 | ATPase activity; actin-filament based movement |
| ANKRD1 | ankyrin repeat domain 1 (cardiac muscle) | -5.1 | 5.7E-03 | R-SMAD binding; RNA polymerase II transcription factor binding |
| PODXL | podocalyxin-like | -4.9 | 2.8E-03 | Positive regulation of cell migration; positive regulation of cell-cell adhesion mediated by integrin |
| RLTPR | RGD motif, leucine rich repeats, tropomodulin domain and proline-rich containing | -4.7 | 1.1E-04 | Cell migration; maintenance of cell polarity |
| DSCAM | Down syndrome cell adhesion molecule | -4.6 | 4.1E-04 | Cell adhesion; positive regulation of phosphorylation |
| ST6GAL2 | ST6 beta-galactosamide alpha-2,6-sialyltranferase 2 | -4.6 | 1.3E-03 | beta-galactoside alpha-2,6-sialyltransferase activity; oligosaccharide metabolic process |
| DUSP2 | dual specificity phosphatase 2 | -4.5 | 4.5E-06 | Protein tyrosine/threonine phosphatase activity; inactivation of MAPK activity |
| LINC00707 | long intergenic non-protein coding RNA 707 | -4.5 | 4.9E-08 | - |
| CXCL8 | chemokine (C-X-C motif) ligand 8 | -4.4 | 3.7E-02 | Chemokine activity; movement of cell and cellular transport |
| | | | | |
| Genes most strongly upregulated with quiescence | | | | |
| C10orf105 | chromosome 10 open reading frame 105 | 7.6 | 2.8E-13 | Integral component of membrane |
| ADH1B | alcohol dehydrogenase 1B (class I), beta polypeptide | 7.3 | 3.0E-03 | Alcohol dehydrogenase activity, zinc-dependent; ethanol oxidation |
| SLC51B | solute carrier family 51, beta subunit | 7.1 | 2.5E-02 | Transporter activity; positive regulation of protein targeting to membrane |
| PPL | periplakin | 7.0 | 3.3E-17 | Structural constituent of cytoskeleton; extracellular exosome |
| COL21A1 | collagen, type XXI, alpha 1 | 6.8 | 3.1E-02 | Extracellular matrix organization |
| ABCA9 | ATP-binding cassette, sub-family A (ABC1), member 9 | 6.4 | 3.7E-08 | ATPase activity coupled to transmembrane movement of substances |
| RASL12 | RAS-like, family 12 | 6.3 | 4.1E-05 | GTP-binding; small GTPase mediated signal transduction |
| WNT11 | wingless-type MMTV integration site family, member 11 | 6.2 | 2.0E-03 | GTPase activator activity; negative regulation of cell growth; negative regulation of cell migration |
| LCNL1 | lipocalin-like 1 | 6.2 | 5.7E-04 | - |
| P2RY14 | purinergic receptor P2Y, G-protein coupled, 14 | 6.1 | 1.5E-02 | UDP-activated nucleotide receptor activity; G-protein coupled receptor signaling pathway |

**Table S3. Differential expression of core splicing factors with quiescence.**

| snRNP | Gene name (alternative name) | log2fold change (7dCI/P) | q-value |
|---|---|---|---|
| U1 | snRNP70 (U1-70K) | -0.702430056 | 0.39522249 |
| U1 | snRPA (U1A) | -0.948210867 | 0.1187233 |
| U1 | snRPC (U1C) | -1.176097682 | 0.03806647 |
| U2 | SF3A1 (SF3a120) | -0.614458322 | 0.61802113 |
| U2 | SF3A2 (SF3a66) | -0.234165381 | 1 |
| U2 | SF3A3 (SF3a60) | -0.873798225 | 0.17495962 |
| U2 | SF3B1 (SF3b155) | 0.312226007 | 0.86175232 |
| U2 | SF3B6 (SF3B14a) | -0.485550585 | 0.55346759 |
| U2 | PHF5A (SF3B14b) | -0.88579857 | 0.17450732 |
| U2 | SF3B2 (SF3b145) | -0.605670987 | 0.52851566 |
| U2 | SF3B3 (SF3b130) | -0.675320143 | 0.26816022 |
| U2 | SF3B4 (SF3b49) | -1.201936526 | 0.14420894 |
| U2 | SF3B5 (SF3B10) | -0.380985328 | 0.68412396 |
| U2 | SNRPB2 | -0.614559179 | 0.49114666 |
| U4/U6 | PRPF3 (hPrp3) | -0.962145763 | 0.14508486 |
| U4/U6 | PRPF31 (hPrp31) | -0.752235842 | 0.23960828 |
| U4/U6 | PRPF4 (hPrp4) | -1.470334425 | 0.00573558 |
| U4/U6 | PPIH (CYPH) | -1.53145057 | 0.00290289 |
| U4/U6 | SNU13 (NHP2L1) | -0.67072951 | 0.32278753 |
| U5 | EFTUD2 (Snu114) | -1.013346266 | 0.07323332 |
| U5 | SnRNP200 (BRR2) | -0.343026961 | 0.79756531 |
| U5 | DDX23 (PRPF28) | -0.790457902 | 0.23178541 |
| U5 | PRPF6 (PRP6) | -0.187170663 | 1 |
| U5 | PRPF8 (PRP8) | -0.470363074 | 0.65113533 |
| U5 | snRNP40 (40K) | -0.914442798 | 0.08016744 |
| U5 | CD2BP2 (52K) | -0.457332647 | 0.60498356 |
| U5 | TXNL4A (DIB1) | -0.843749525 | 0.21580353 |
| U4/U5/U6 only | SART1 (hSnu66) | -0.810010974 | 0.33443671 |
| U4/U5/U6 only | BRSK1 (hSAD1) | 0.287152548 | 0.79841598 |
| U4/U5/U6 only | SNRNP27 (27K) | -0.63082609 | 0.42316184 |

The significant genes (q-value < 0.05) are highlighted in yellow.

**Table S4. Number of RNA-Seq reads for Polyadenylation Site-enriched RNA-Sequencing.**

The number of reads for each sample in each Illumina sequencing run after mapping to the human genome (hg19) using the Tophat alignment algorithm are provided. Table also includes the number of reads that were retained in the analysis after assigning them to polyadenylation sites (pA) from human PolyAsite annotation (http://www.polyasite.unibas.ch/).

| | Proliferating (P) | | 7 day Contact Inhibited (7dCI) | |
|---|---|---|---|---|
| Biological Replicate | 12-1 | 12-3 | 12-1 | 12-2 |
| Total Number of reads mapped to hg19 uisng Tophat | $0.830 \times 10^7$ | $0.678 \times 10^7$ | $0.788 \times 10^7$ | $0.699 \times 10^7$ |
| Percent of reads mapped to hg19 using Tophat | 81.10% | 81.74% | 79.52% | 78.89% |
| Reads mapping to pA sites from PolyAsite annotation | $0.607 \times 10^7$ | $0.498 \times 10^7$ | $0.571 \times 10^7$ | $0.491 \times 10^7$ |
| Percentage of reads mapping to pA sites from PolyAsite annotation | 62.92% | 63.51% | 61.90% | 59.54% |

| | Control siRNA | | CstF64 siRNA | | CPSF73 siRNA | | CFIm25 siRNA | |
|---|---|---|---|---|---|---|---|---|
| Biological Replicate | 12-1 | 12-3 | 12-1 | 12-3 | 12-1 | 12-3 | 12-1 | 12-3 |
| Total Number of reads mapped to hg19 uisng Tophat | $0.864 \times 10^7$ | $0.944 \times 10^7$ | $0.920 \times 10^7$ | $0.611 \times 10^7$ | $0.937 \times 10^7$ | $0.912 \times 10^7$ | $0.931 \times 10^7$ | $0.755 \times 10^7$ |
| Percent of reads mapped to hg19 using Tophat | 81.30% | 79.25% | 82.27% | 84.22% | 78.94% | 80.99% | 80.03% | 80.17% |
| Reads mapping to pA sites from PolyAsite annotation | $0.646 \times 10^7$ | $0.698 \times 10^7$ | $0.702 \times 10^7$ | $0.472 \times 10^7$ | $0.702 \times 10^7$ | $0.684 \times 10^7$ | $0.707 \times 10^7$ | $0.557 \times 10^7$ |
| Percentage of reads mapping to pA sites from PolyAsite annotation | 63.83% | 60.77% | 66.39% | 67.01% | 62.27% | 62.26% | 63.65% | 60.41% |

| | P, time=0hr | | P, time=2hr | | P, time=4hr | | P, time=8hr | |
|---|---|---|---|---|---|---|---|---|
| Biological Replicate | 12-1 | 12-3 | 12-1 | 12-3 | 12-1 | 12-3 | 12-1 | 12-3 |
| Total Number of reads mapped to hg19 uisng Tophat | $0.958 \times 10^7$ | $0.882 \times 10^7$ | $1.040 \times 10^7$ | $0.947 \times 10^7$ | $0.899 \times 10^7$ | $1.107 \times 10^7$ | $1.210 \times 10^7$ | $1.275 \times 10^7$ |
| Percent of reads mapped to hg19 using Tophat | 81.38% | 80.95% | 80.89% | 81.48% | 82.12% | 80.75% | 79.45% | 78.42% |
| Reads mapping to pA sites from PolyAsite annotation | $0.624 \times 10^7$ | $0.559 \times 10^7$ | $0.689 \times 10^7$ | $0.615 \times 10^7$ | $0.601 \times 10^7$ | $0.724 \times 10^7$ | $0.787 \times 10^7$ | $0.825 \times 10^7$ |
| Percentage of reads mapping to pA sites from PolyAsite annotation | 65.16% | 63.36% | 66.24% | 64.88% | 66.90% | 65.43% | 65.05% | 64.72% |
| | 7dCI, time=0hr | | 7dCI, time=2hr | | 7dCI, time=4hr | | 7dCI, time=8hr | |
| Biological Replicate | 12-1 | 12-3 | 12-1 | 12-3 | 12-1 | 12-3 | 12-1 | 12-3 |
| Total Number of reads mapped to hg19 uisng Tophat | $0.928 \times 10^7$ | $0.745 \times 10^7$ | $0.809 \times 10^7$ | $1.014 \times 10^7$ | $1.015 \times 10^7$ | $1.237 \times 10^7$ | $1.162 \times 10^7$ | $1.425 \times 10^7$ |
| Percent of reads mapped to hg19 using Tophat | 80.69% | 80.53% | 80.34% | 79.38% | 80.47% | 78.84% | 77.85% | 77.82% |
| Reads mapping to pA sites from PolyAsite annotation | $0.606 \times 10^7$ | $0.475 \times 10^7$ | $0.531 \times 10^7$ | $0.664 \times 10^7$ | $0.679 \times 10^7$ | $0.825 \times 10^7$ | $0.735 \times 10^7$ | $0.920 \times 10^7$ |
| Percentage of reads mapping to pA sites from PolyAsite annotation | 65.27% | 63.71% | 65.64% | 65.51% | 66.94% | 66.71% | 63.31% | 64.60% |

**Table S5. Expression levels of factors that bind motifs present in the alternate regions of quiescence-induced longer isoforms**

| Gene | log2fold(7dCI/P) | pvalue | padj |
|---|---|---|---|
| SNRPA | -1.937944049 | 3.83E-15 | 2.36E-13 |
| SRSF2 | -1.6088995 | 2.16E-16 | 1.65E-14 |
| SRSF7 | -1.596246825 | 2.83E-15 | 1.78E-13 |
| ELAVL1 | -1.25314909 | 1.44E-07 | 2.48E-06 |
| FUS | -1.232860781 | 3.99E-06 | 4.97E-05 |
| PPRC1 | -1.156598769 | 0.00931581 | 0.03855354 |
| DAZAP1 | -1.087951629 | 7.93E-05 | 6.94E-04 |
| U2AF2 | -1.031617616 | 4.07E-05 | 3.85E-04 |
| SRSF1 | -0.987796533 | 1.04E-05 | 1.17E-04 |
| RBM8A | -0.950889009 | 6.21E-07 | 9.21E-06 |
| HNRNPL | -0.727136968 | 0.30577249 | 0.51091149 |
| RBM28 | -0.614668732 | 0.11006761 | 0.25354424 |
| RBM42 | -0.606102322 | 0.01922101 | 0.06813312 |
| HNRNPA1 | -0.539541149 | 0.00294536 | 0.01513658 |
| G3BP2 | -0.497526883 | 0.08199056 | 0.2053361 |
| YBX1 | -0.457655514 | 0.02880999 | 0.09323427 |
| PCBP1 | -0.368999482 | 0.06552294 | 0.17280009 |
| FXR2 | -0.323622195 | 0.51141416 | 0.70244058 |
| HNRNPA2B1 | -0.21895066 | 0.36383014 | 0.57145414 |
| RBM4 | -0.200712814 | 0.7201741 | 0.84944376 |
| RALY | -0.18879302 | 0.31839036 | 0.52493248 |
| SAMD4A | -0.061618966 | 0.88955756 | 0.94763983 |
| ANKHD1 | -0.036773719 | 0.94566291 | 0.97514581 |
| TIA1 | 0.051108965 | 0.84367583 | 0.92183425 |
| QKI | 0.111685664 | 0.70254596 | 0.83800393 |
| CNOT4 | 0.192246741 | 0.71242501 | 0.84487501 |
| CPEB2 | 0.195566126 | 0.78374536 | 0.88759547 |
| CPEB4 | 0.236009501 | 0.73496651 | 0.8585516 |
| HNRNPH2 | 0.541088962 | 0.01875685 | 0.06683603 |
| FMR1 | 0.632320832 | 0.24979597 | 0.44752334 |
| PCBP2 | 0.724951786 | 4.17E-04 | 0.00292084 |
| RBM5 | 0.741169877 | 0.0059837 | 0.02714546 |
| RBM6 | 1.053632478 | 0.00135285 | 0.0078863 |
| ZC3H10 | 1.583864929 | 0.01978887 | 0.06973454 |
